# Controlled Aggregation of Citrate-Stabilized Gold Nanoparticles into Long-Lived Metastable Clusters

**DOI:** 10.1021/acs.langmuir.6c03197

**Published:** 2026-09-12

**Authors:** Maelly A. Oliveira, Rômulo A. Ando

**Affiliations:** Laboratory of Molecular Spectroscopy, Department of Fundamental Chemistry, Institute of Chemistry, University of São Paulo, Avenida Professor Lineu Prestes, 748, São Paulo, São Paulo CEP 05508-000, Brazil

## Abstract

The controlled aggregation of plasmonic nanoparticles remains a critical challenge for achieving temporally stable and reproducible colloidal substrates for surface-enhanced Raman scattering (SERS). Here, we show that the antihistamine drug bilastine induces a concentration-dependent, self-limiting aggregation of citrate-capped gold nanoparticles (cit-AuNPs), producing long-lived metastable clusters in aqueous media. UV–Vis spectroscopy reveals the emergence and temporal evolution of a lower-energy, plasmon-coupling band, while dynamic light scattering (DLS) supports the formation of larger colloidal structures in suspension. Transmission electron microscopy (TEM) provides complementary qualitative evidence of concentration dependent changes in aggregate morphology. Zeta potential measurements show progressive interfacial charge regulation upon bilastine addition, consistent with perturbation of the citrate-stabilized interface. SERS spectra and citrate-control experiments support the presence of bilastine in the SERS-active interfacial region, with enhanced carboxylate-associated modes suggesting proximity of the carboxylate-containing region to the Au surface. Time-dependent optical measurements and semi-quantitative DLVO-type analysis indicate a slowly evolving, self-limiting aggregation regime at selected submicromolar bilastine concentration, whereas higher concentrations promote faster destabilization. The resulting aggregates retain SERS activity over extended periods, highlighting bilastine as a molecular modulator for generating long-lived SERS-active AuNP clusters.

## Graphical abstract

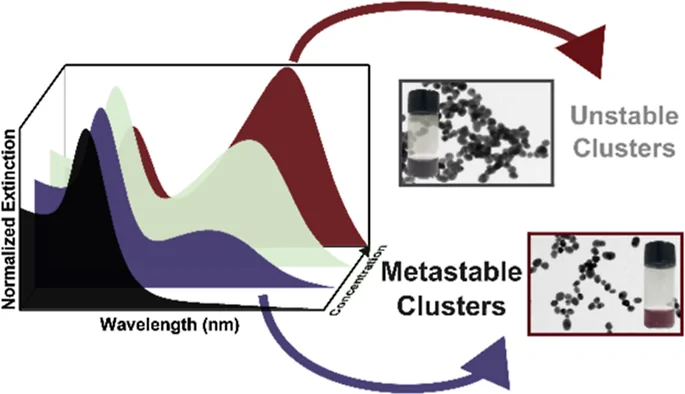

## 1 Introduction

The unique optical, electrical, and chemical properties of plasmonic nanoparticles attract widespread interest across multiple fields, such as targeted drug delivery, biosensing, bioimaging, and catalytic degradation.^1–5^ In colorimetric sensing, the characteristic coloration of metallic colloids arises from localized surface plasmon resonance (LSPR), which represents the collective oscillation of geometrically confined conduction electrons.^3,6–12^ Because LSPR is highly sensitive to particle size, shape, composition, and local environment, subtle changes in these parameters can produce distinct spectral shifts and color variations.^3,6^ While many applications require colloidal stabilization against aggregation, controlled particle association can be advantageous in other contexts, particularly in surface enhanced Raman scattering (SERS).^13^ The interaction of incident electromagnetic radiation with plasmonic nanostructures generates strong local electromagnetic-field enhancement near the metal surface, which is further amplified when two or more particles are brought into close proximity, producing interparticle hotspots.^6,14^ Because SERS enhancement scales sharply within these intensified near-fields, hotspot generation is critical for sensitive molecular detection.^6^ However, aggregates formed through conventional self-assembly are often random, dynamically unstable, and poorly reproducible, limiting their use in nanoaggregate-based SERS applications.^15,16^

To address the limited reproducibility of randomly formed colloidal aggregates, bottom-up self-assembly strategies using molecular and supramolecular agents have emerged as practical alternatives to nanofabrication.^17–22^ Amino acids and DNA-based structures can mediate controlled interactions between neighboring particles, while supramolecular linkers, such as cucurbiturils, can generate precise subnanometer gaps that provide excellent analytical SERS reproducibility.^21–30^ Imidazole-based compounds are also known to promote nanoparticle assembly effectively, which has directed attention toward related systems, including ionic liquids (ILs).^31,32^ Furthermore, some rationally designed assembly strategies exhibit reversibility, where external stimuli such as temperature, light and pH changes trigger dissociation.^33–38^

Although these approaches control interparticle spacing and hotspot formation, many primarily address the initial assembly geometry rather than their long-term temporal evolution. Consequently, the kinetic persistence of the resulting assemblies remains an important challenge, as colloidal aggregates can continue to evolve toward irreversible coagulation and precipitation.^23,39^ Recent studies have therefore focused on kinetic-arrest or self-limiting assembly strategies capable of preserving plasmonic hotspots over extended periods.^23,39–42^ In kinetic-arrest approaches, cluster growth is halted by introducing an additional stabilizing component after assembly has begun, thereby suppressing further cluster growth while preserving hotspot architecture. Potter et al., for example, demonstrated kinetic arrest of cucurbituril-induced gold nanoparticle aggregates using poly­(ethylene glycol) encapsulation.^23^ In another approach, Namigata et al. entrapped salt-induced aggregates within polyacrylamide hydrogel frameworks, preventing continuous clustering.^42^ In contrast, self-limiting strategies avoid secondary agents by tuning intrinsic balance of interparticle interactions. Accordingly, Meyer et al. showed that adjustment of KCl concentration can modulate the aggregation of silver colloids, producing small clusters through a Coulomb-blocking mechanism that remained stable for periods exceeding six months.^40^

Currently reported strategies typically rely either on secondary stabilizing components or on careful adjustment of suspension conditions, including ionic strength. In addition to the practical drawback of adding auxiliary compounds to the sample, salt-induced aggregation often provides limited control over aggregate morphology. Accordingly, molecular agents capable of promoting nanoparticle assembly through specific metal-molecule or surface interactions while simultaneously limiting further aggregate growth represent an attractive strategy for persistent colloidal SERS substrates.

Among the systems explored for controlled aggregation of gold nanoparticles, biologically active molecules represent a particularly interesting class of assembly agents.^19,26,43–46^ In this context, synthetic drugs remain comparatively unexplored despite their structural diversity and abundance of functional groups, which may provide opportunities for tuning nanoparticle interfacial chemistry while also offering insight into the behavior of pharmaceutical compounds at plasmonic surfaces. Bilastine, a second generation antihistamine, is a promising molecular candidate because it contains multiple ionizable and interfacially active groups, including amine-containing moieties and a terminal carboxylate group. These structural features may enable bilastine to associate with citrate-capped gold nanoparticles, modulate their surface charge, and influence the temporal evolution of the resulting aggregates. In this work, we investigate the ability of bilastine to induce concentration-dependent, self-limiting aggregation of citrate-capped gold nanoparticles. We show that bilastine can generate long-lived, SERS-active metastable aggregates in aqueous media and discuss how its multifunctional molecular structure contributes to interfacial charge regulation and aggregate persistence.

## 2 Experimental Section

### 2.1 Materials

Gold­(III) chloride trihydrate (HAuCl_4_·3H_2_O ≥ 99%) and sodium citrate dihydrate (Na_3_C_6_H_5_O_7_·2H_2_O, ≥99%) were purchased from Sigma-Aldrich and used without further purification. Ultrapure Milli-Q water was used throughout all experiments.

### 2.2 Gold Nanoparticle Synthesis (Cit-AuNP)

Citrate-stabilized gold nanoparticles were synthesized using a standard Turkevich procedure.^47^ Briefly, 72.7 mL of Milli-Q water and 24.3 mL of 2.5 mM HAuCl_4_ were added to a 125 mL Erlenmeyer flask previously cleaned with aqua regia (HCl/HNO_3_ 3:1 v/v). The mixture was heated under vigorous stirring to approximately 90°C, after which 3.0 mL of a preheated 1% sodium citrate solution (0.1 g/10 mL) was added. Heating and stirring were maintained for at least 15 min, during which the solution became reddish-purple. The synthesis yielded cit-AuNPs with an average diameter of 39.6 ± 8 nm (Figure S1 in Supporting Information), an estimated concentration of 3 × 10^10^ NPs mL^–1^, and pH of 5.^48^ The nanoparticles were stored at room temperature and protected from light until use, without further purification. The nominal concentrations of excess citrate and other ionic species estimated from the synthetic procedure are listed in Table S1.

### 2.3 Preparation of Bilastine Solutions

Bilastine stock solution (500 μM) was prepared in Milli-Q water. From this stock, aqueous bilastine solutions with concentrations of 1, 2.5, 5, 7.5, 10, 20, 30, 40, 50, 60, 70, 80, 100, 250, and 500 μM were prepared by dilution. The upper concentration limit was defined based on the reported aqueous solubility of bilastine, approximately 0.3 mg mL^–1^. These solutions were mixed with cit-AuNPs to obtain final bilastine concentrations in the range of 0.10–10 μM.

### 2.4 UV–Vis Extinction Spectroscopy

UV–Vis extinction spectra were recorded using a Shimadzu UV-3101PC spectrophotometer and quartz cuvettes with 5 mm optical path length. Cit-AuNPs and bilastine solutions were mixed in appropriate volumes to give a final volume of 1500 μL while maintaining the desired final bilastine concentration. Unless otherwise stated, spectra were recorded immediately after mixing.

### 2.5 Surface-Enhanced Raman Scattering (SERS)

SERS spectra were acquired using a Renishaw InVia Raman Microscope equipped with a 785 nm laser. Measurements were performed with 15 mW laser power at the sample and an Olympus 20x long-working-distance objective. For each sample, 980 μL of cit-AuNP suspension was mixed with 20 μL of bilastine solution to obtain the desired final bilastine concentration. Aliquots of 200 μL were transferred to Eppendorf tube caps used as sample holders. Spectra were acquired with an integration time of 10s and 3 accumulations.

### 2.6 Transmission Electron Microscopy (TEM)

TEM images of cit-AuNPs and cit-AuNP/bilastine mixtures were obtained using a JEOL JEM 2100 microscope. The mixtures were prepared using the same procedure described for SERS experiments and incubated for approximately 2 min before deposition. After the required dilutions, 5 μL aliquots were drop-cast onto Formvar/carbon-coated 200 Mesh copper grids and dried at room temperature.

### 2.7 Dynamic Light Scattering (DLS) and Zeta Potential Measurements

DLS and ζ-potential measurements were performed using a Malvern Zetasizer Nano ZS equipped with a 633 nm He–Ne laser. The samples were prepared using the same proportions described for the SERS measurements and equilibrated at 25°C for a few minutes before analysis. Each result corresponds to the average of at least three measurements. DLS data are reported as intensity-weighted hydrodynamic diameter distributions. ζ-potential values were obtained from electrophoretic mobility measurements using the Smoluchowski approximation.

## 3 Results and Discussion

### 3.1 Bilastine-Induced Gold Nanoparticle Assembly

The interaction between bilastine and citrate-stabilized gold nanoparticles prepared by the Turkevich method was initially investigated by UV–Vis extinction spectroscopy. Immediately after mixing bilastine with the gold colloid, a distinct lower-energy band becomes optically resolvable at bilastine concentrations above 0.4 μM (Figure 1A), indicating the formation of plasmonically coupled nanoparticle populations.

**Figure 1:**
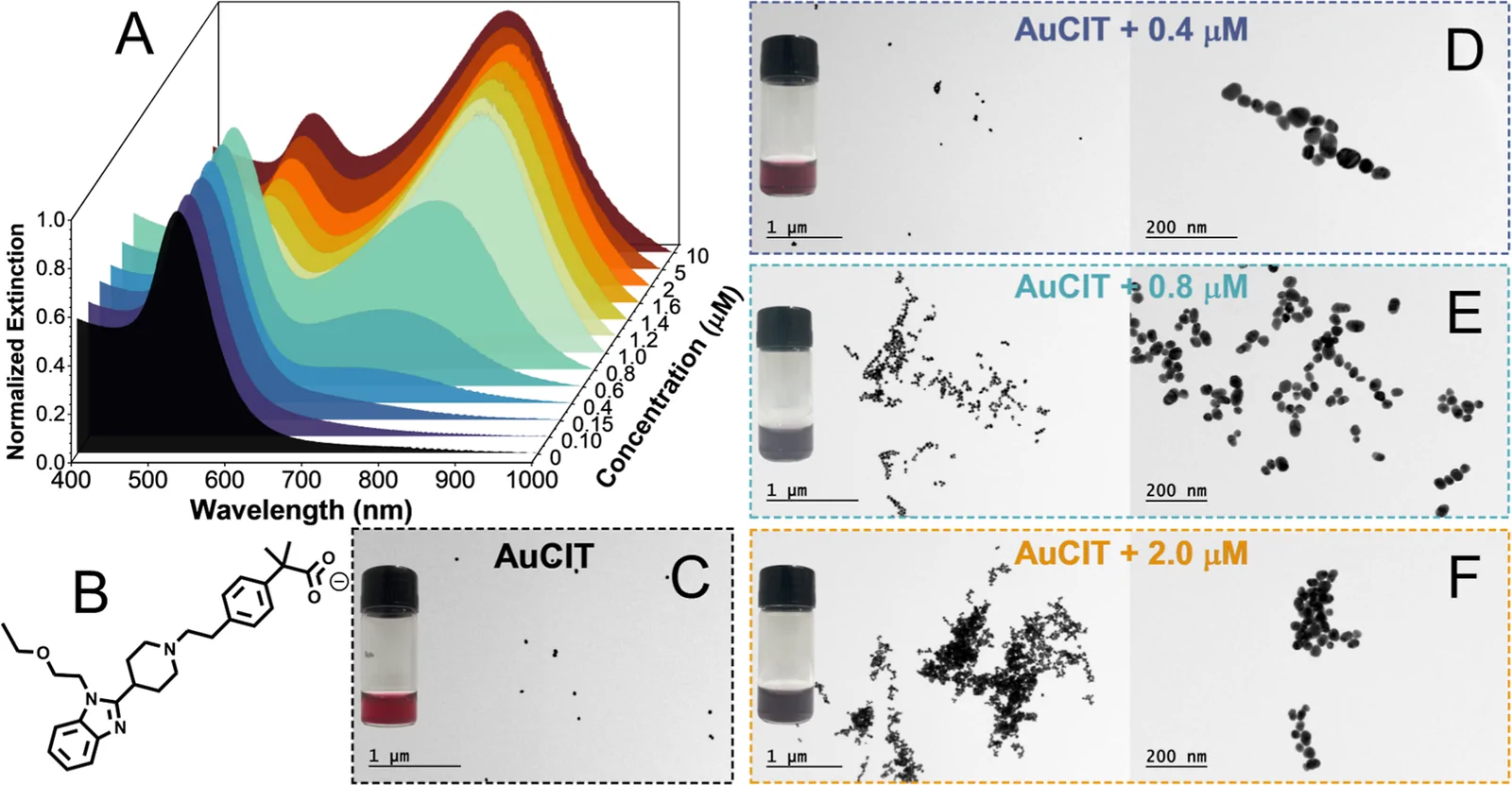
(A) UV–Vis extinction spectra of cit-AuNPs after addition of different bilastine concentrations. (B) Molecular structure of bilastine. Representative TEM images of (C) cit-AuNPs and cit-AuNPs/bilastine mixtures containing (D) 0.4 μM, (E) 0.8 μM and (F) 2.0 μM bilastine.

The emergence of this lower energy band is consistent with electromagnetic coupling between neighboring AuNPs brought into close proximity. Such coupling is often observed in anisotropic assemblies, including chain-like aggregates, where collective oscillation of conduction electrons can give rise to a longitudinal plasmon mode at longer wavelengths. A related optical behavior is observed in gold nanorods, in which structural elongation breaks the degeneracy of electron oscillations and produces two plasmon modes: a transverse mode at higher energy and a longitudinal mode at lower energy.^49,50^ However, the optical spectrum alone does not uniquely determine aggregate morphology. Therefore, the lower energy band is interpreted here as evidence for the formation of plasmonically coupled nanoparticle aggregates, with possible contributions from elongated, branched, compact, and coupled structures. This interpretation is consistent with previous studies on molecularly induced AuNP aggregation, particularly in systems involving amino acids.^25,51^ Theoretical studies have shown that the redshift of the longitudinal plasmon band generally correlates with increasing chain length and decreasing interparticle spacing, whereas the spectral profile, including band shape and width, is sensitive to variation in aggregate geometry.^50,52,53^

As observed in Figure 1A, the lower energy plasmon band becomes progressively more defined as the bilastine concentration increases, showing higher intensity and narrower bandwidth up to approximately 1 μM. Concurrently, the band maximum redshifts up to this concentration, followed by a blueshift at higher bilastine concentrations. The redshift observed up to 1 μM is consistent with increasing interparticle coupling and growth of the aggregate population. At higher concentrations, the blueshift suggests a change in the dominant aggregate morphology and/or in the relative population of optically active aggregates. Two non-exclusive effects may contribute to this behavior. First, at higher concentrations, faster aggregation may change the relative contribution of optically active aggregate populations, so that the lower energy plasmon band no longer follows a simple monotonic relationship with aggregate size. Second, excess bilastine adsorbed or co-adsorbed at the nanoparticle interface may modify short-range interparticle interactions, hindering further elongation of intermediate-sized aggregates.^54,55^

It is important to emphasize that the bimodal extinction profile does not imply the exclusive formation of rod-like or linear aggregates. The band near the original transverse plasmon resonance includes contributions from dispersed or weakly coupled AuNPs, whereas the lower energy band reflects plasmonic coupling within the aggregated nanoparticle population. Thus, the UV–Vis spectra indicate the coexistence of dispersed nanoparticles and coupled aggregates in solution, with their relative populations and morphologies varying as a function of bilastine concentration.

Transmission electron microscopy (TEM) images were obtained for selected bilastine concentrations (Figure 1D–F). Because conventional TEM requires sample drying, these images should be interpreted as qualitative complementary evidence rather than a direct representation of the aggregate distribution in suspension. At 0.4 μM bilastine, isolated nanoparticles and small aggregates are observed, consistent with the weak and broad lower energy band in the UV–Vis spectrum. Figure S2 (Supporting Information) shows representative TEM images illustrating few chain-like aggregates. At 0.8 μM, elongated and branched aggregates become more frequent, supporting the formation of anisotropic coupled structures. At 2 μM, larger and more irregular aggregates are observed, in agreement with the faster aggregation and precipitation tendency at higher bilastine concentrations. Notably, Figure 1E,F, as well as Figures S3 and S4 (Supporting Information), show the contribution of chain-like aggregates that persist under these higher concentrations.

Dynamic light scattering (DLS) measurements (Figure S5) provide solution-phase evidence for bilastine-induced aggregation and complement the optical and TEM results. The intensity-weighted hydrodynamic-diameter distributions of pristine cit-AuNPs show a main population centered at approximately 45.8 nm. Upon addition of low bilastine concentrations, the larger population remains close to that of the original colloid, with only minor shifts to approximately 46.0, 49.6, and 54 nm for 0.10, 0.15, and 0.40 μM bilastine, respectively. These modest changes indicate that, below 0.4 μM, most nanoparticles remain dispersed or only weakly associated, although the small increase in apparent hydrodynamic diameter is consistent with the initial formation of a limited population of small aggregates. A more pronounced change is observed at 0.8 μM, where the distribution becomes polydisperse and larger scattering populations appear (larger population at 1169 nm), including aggregates in the submicrometer to micrometer range. This abrupt change supports that bilastine-induced aggregation occurs in aqueous suspension and not only during drying for TEM analysis.

At higher bilastine concentrations (1–5 μM), DLS measurements show that the intensity-weighted distributions remain dominated by large scattering populations near the micrometer range. Because these samples are highly polydisperse and prone to sedimentation, the hydrodynamic diameters should be interpreted qualitatively rather than as precise aggregate-size distributions. Together with the UV–Vis spectra and TEM images, these results indicate that increasing bilastine concentration promotes larger and more heterogeneous aggregate populations. The apparent blueshift of the lower-energy plasmon band at high bilastine concentration is therefore attributed to changes in the relative contribution of optically active aggregate populations rather than to a simple monotonic decrease or increase in aggregate size.

### 3.2 Interfacial Charge Regulation and Bilastine Association at the AuNP Interface

To evaluate how bilastine modifies the electrokinetic properties of the nanoparticles, the zeta potential (ζ) of the colloid was monitored as a function of bilastine concentration (Figure 2A). Although ζ-potential does not directly correspond to the true surface potential, it provides an important descriptor of the electrostatic environment near the slipping plane and is useful for following interfacial charge regulation in colloidal suspensions. In general, colloids with |ζ| ≥30 mV are considered electrostatically stable, whereas systems with |ζ| values in the range of approximately 10–15 mV exhibit only incipient electrostatic stabilization.^7^ In the present system, the ζ-potential evolves from approximately −32 mV for cit-AuNPs to positive values as the bilastine concentration increases at constant ionic strength (Table S1 in Supporting Information), passing through the incipient stability regime at intermediate concentrations. This trend is consistent with the concentration-dependent aggregation behavior observed by UV–Vis spectroscopy, TEM and DLS.

**Figure 2:**
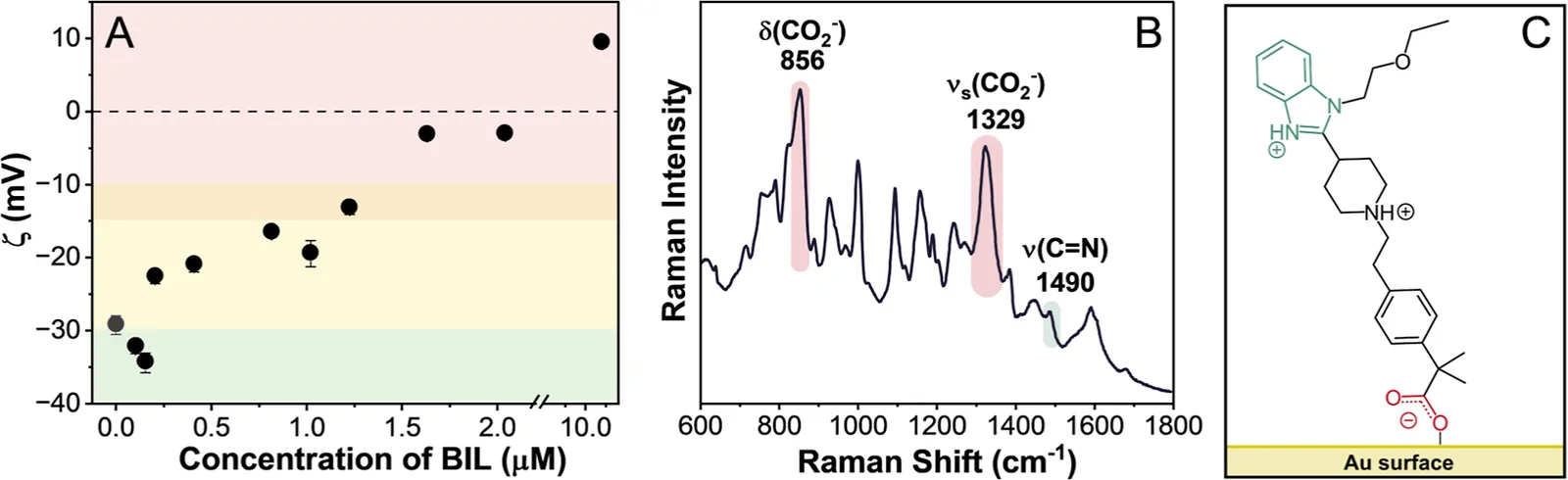
(A) Concentration-dependent ζ-potential of cit-AuNPs/bilastine mixtures. The colored regions indicate approximate colloidal stability regimes based on the magnitude of ζ-potential. (B) SERS spectrum of a 0.8 μM aqueous bilastine solution in the presence of cit-AuNPs, with highlighted vibrational modes associated with the carboxylate and benzimidazole moiety. (C) Proposed schematic representation of bilastine association at the AuNP interface, consistent with the SERS response and ζ-potential evolution.

The ζ-potential response indicates that bilastine substantially modifies the nanoparticle interface. At very low bilastine concentrations, 0.1 and 0.15 μM, the ζ-potential becomes slightly more negative (Figure 2A). This behavior could at first suggest enhanced electrostatic stabilization, however, subtle changes in the long-wavelength region (700–800 nm) of the extinction spectra and the modest increase in hydrodynamic diameter observed by DLS indicate the formation of only a small population of weakly coupled aggregates. Because ζ-potential measurements provide an ensemble-averaged electrokinetic response, they are expected to be dominated by the larger fraction of dispersed or weakly associated nanoparticles.^7^ A possible interpretation is that low bilastine surface coverage locally perturbs the citrate layer, counterion distribution, and hydration environment near the slipping plane, producing a slightly more negative apparent ζ-potential while only a limited fraction of particles undergoes aggregation.

At higher bilastine concentrations, the progressive increase in ζ and eventual transition to positive values indicate a substantial interfacial charge regulation. Since the amount of bilastine added is small compared with the nominal ionic background of the as-synthesized colloid, nonspecific compression of the electrical double layer by bulk ionic strength increase alone is unlikely to account for observed behavior. Instead, the data are more consistent with bilastine adsorption or co-adsorption at the citrate stabilized AuNP interface, accompanied by partial neutralization and perturbation of the negatively charged citrate layer. Importantly, ζ-potential measurements alone cannot distinguish complete citrate replacement from mixed citrate/bilastine interfacial coverage. Therefore, the observed charge inversion should be interpreted as evidence of interfacial charge regulation rather than a direct proof of a complete ligand-exchange process.^56^ The pH-dependent experiments further support the role of bilastine protonation in the aggregation process. Literature pK_a_ values for the carboxylate (p*K*_a_∼3) and piperidine (p*K*_a_ ∼9) groups suggest that bilastine is expected to be predominantly zwitterionic near the working pH.^57,58^ However, the UV–Vis pH dependent experiments (Figure S6 and Scheme S1) suggest that benzimidazole moiety may also participate in the protonation equilibrium, with an apparent pK_a_ in the range of approximately 5–7. Thus, under the experimental conditions, a fraction of bilastine molecules may contain protonated sites capable of electrostatic association with the negatively charged citrate-stabilized AuNP surface. Consistent with this interpretation, increasing the suspension pH suppresses aggregation (Figure S7), likely because bilastine speciation shifts away from protonated forms that can effectively interact with the citrate coated interface. Moreover, no significant bulk pH changes were observed after bilastine addition to the gold colloid, indicating that the aggregation behavior is not simply caused by pH variation of the medium.

To obtain additional spectroscopic evidence for bilastine association with the AuNP interface, SERS spectra of bilastine were recorded in the presence of cit-AuNPs (Figure 2B). Control SERS spectra of pristine cit-AuNPs and KCl-aggregated cit-AuNPs (Figure S8) show only weak citrate-related features, which appear at different wavenumbers and do not reproduce the spectral profile observed for the bilastine-containing aggregates. Therefore, the bands observed in Figure 2B are assigned primarily to bilastine rather than to residual citrate. The presence of bilastine in the SERS-active interfacial region is supported by the overall vibrational fingerprint, including bands associated with its aromatic/heteroaromatic and piperidine-containing framework, together with the enhanced carboxylate-associated modes. The strong relative enhancement of bands assigned to the bending, δ­(CO_2_^–^), and symmetric stretching, ν_s_(CO_2_^–^), modes, is consistent with proximity of the carboxylate-containing region to the Au surface, as expected from the distance dependence of SERS enhancement and from surface-selection-rule considerations. This interpretation is also chemically plausible in light of previous reports describing the interaction of carboxylate-containing molecules with gold surfaces.^6,14,59^ Nevertheless, SERS intensities are influenced not only by molecule–surface distance, but also by molecular orientation, local-field polarization, chemical enhancement, protonation state, and hotspot heterogeneity. Therefore, these spectral changes support bilastine adsorption or co-adsorption, as schematically represented in Figure 2C, but do not prove complete citrate displacement or uniquely determine the adsorption geometry.

### 3.3 Time-Dependent Aggregate Evolution and Interaction-Potential Analysis

The temporal evolution of the lower energy plasmon band provides further insight into the kinetics of bilastine induced aggregation (Figure 3A). For all selected bilastine concentrations, the plasmon band observed in the 700–900 nm region shifts progressively over the 60 min monitoring period, with the most pronounced changes occurring shortly after mixing. This behavior indicates that nanoparticle association begins rapidly upon bilastine addition, but that aggregated population continues to evolve over time rather than becoming instantaneously arrested in a static configuration. The absence of a clear plateau within the first hour is consistent with a dynamic aggregation process in which cluster growth, reorganization, and changes in the relative population of coupled structures continue after the initial particle association step. The magnitude and final position of the redshifted plasmon band depend strongly on bilastine concentration. At intermediate concentrations, particularly around 1 μM, the plasmon response reaches the longest wavelengths, indicating stronger plasmonic coupling within the aggregated population. At higher concentrations, such as 2 and 10 μM, it appears at lower wavelengths despite continued temporal evolution. This behavior is consistent with the formation of larger and more heterogeneous aggregate populations, as supported qualitatively by the combined UV–Vis, DLS, and TEM results.

**Figure 3:**
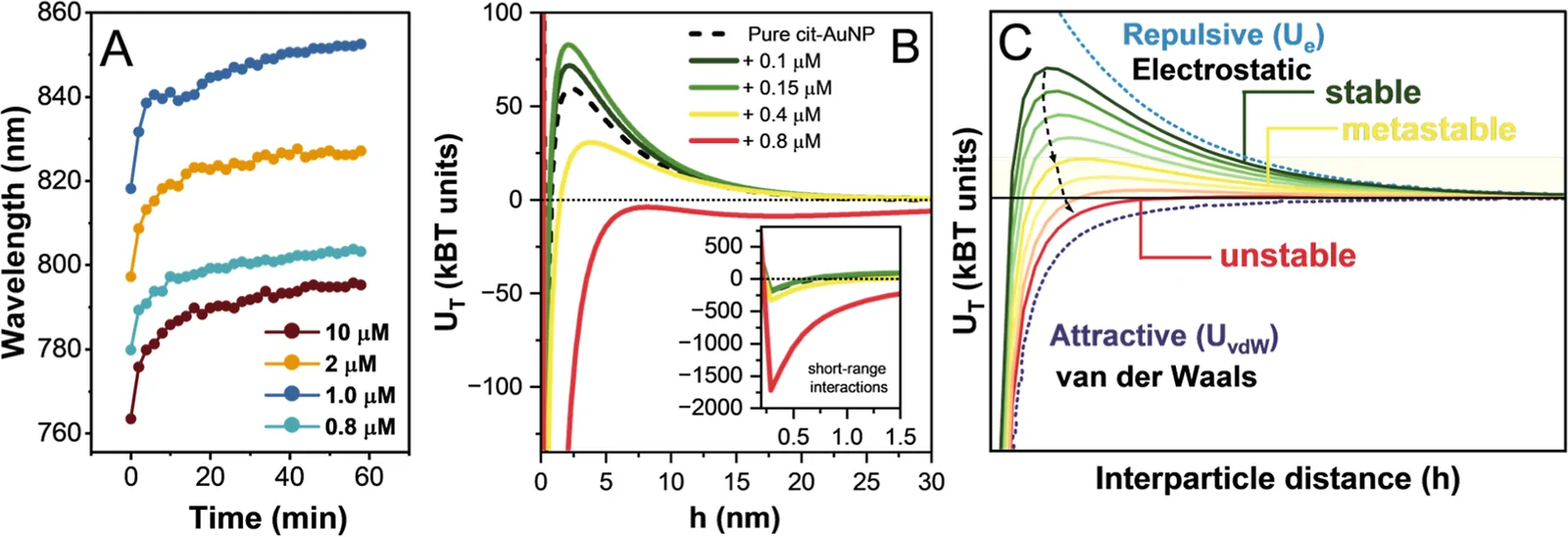
(A) Time-dependent shift of the lower energy plasmon mode over 60 min for selected cit-AuNP/bilastine mixtures. Spectra were recorded every 2 min. (B) Approximate DLVO-type interaction potentials calculated for selected bilastine concentrations. The inset shows the short-range interaction region, including the finite primary minimum generated by the Born-type repulsive contribution. (C) Schematic representation of the qualitative evolution of the interaction-potential profile as the concentration of aggregation modulator increases. Born-type repulsive interactions are omitted in panel C for clarity.

To place this concentration-dependent behavior within a colloidal-interaction framework, selected systems were analyzed using an approximate DLVO-type interaction-potential model (Figure 3B). The total interaction potential was estimated by considering van der Waals attraction, electrostatic double-layer repulsion and a short-range Born-type repulsive contribution.^7,60–63^ Details of the model, parameters, and assumptions are provided in the Supporting Information (Section S.5). Because quantities such as the true surface potential, Hamaker constant, free citrate concentration, complete ionic composition, and molecular-scale steric or hydration contributions from adsorbed bilastine are not independently known, the calculated curves should be regarded as semi-quantitative estimates rather than exact predictions of aggregation kinetics.^40^ Despite these limitations, the calculated interaction potentials capture the expected qualitative trend. For cit-AuNPs, a substantial repulsive barrier is obtained, consistent with electrostatic stabilization of the citrate coated colloid. At very low bilastine concentrations, 0.10 and 0.15 μM, the calculated barriers remain high, indicating that most particles should remain dispersed or weakly associated. At 0.40 μM, the barrier decreases markedly but remains positive, which is consistent with particle approach becoming more favorable while rapid uncontrolled coagulation is still kinetically hindered. At 0.80 μM, the calculated repulsive barrier disappears, producing an attractive interaction profile and predicting a transition toward faster aggregation.

The short-range region of the calculated potential also suggests that, once particles overcome the initial barrier and enter the primary minimum, redispersion is not favored under the investigated conditions. This behavior contrasts with reversible systems characterized by shallow interaction wells, such as the thermoresponsive assembly reported by Liu et al.^38^ Consistently, TEM images of the one-month-aged cit-AuNP/bilastine system show coalescence between adjacent nanoparticles (Figure S9), qualitatively supporting the interpretation that particle association becomes effectively irreversible after close contact is established.

Overall, this trend agrees qualitatively with the experimental observation that bilastine progressively destabilizes the citrate-stabilized AuNP interface. However, the model does not fully reproduce all experimental features. For example, weak aggregation signatures are already observed at the lowest bilastine concentrations, despite the high calculated barriers, and some aggregated structures persist experimentally even under conditions for which the model predicts strong instability. These discrepancies indicate that bilastine induced aggregation is not governed by classical electrostatic and van der Waals interactions alone. Additional contributions associated with bilastine adsorption or co-adsorption, including hydration, steric effects, acid–base interactions, hydrophobic interactions, and interfacial heterogeneity, are likely important in determining the observed aggregation kinetics.

The schematic interaction-potential diagram in Figure 3C summarizes the qualitative transition from a stable colloid, where a large electrostatic barrier prevents particle approach, to a metastable regime, where the barrier is partially reduced and controlled association becomes possible, and finally to an unstable regime, where attractive interactions dominate, and rapid aggregation is favored. In the present system, bilastine appears to move the colloid along this trajectory by regulating the electrokinetic interface and modifying short-range interparticle interactions. Thus, the combined time-dependent extinction and DLVO-type analyses support a dynamic self-limiting aggregation mechanism: bilastine initially reduces the repulsive barrier and promotes particle association, while molecular and interfacial effects slow further growth. This self-limiting aggregation then allows SERS-active aggregates to persist over experimentally relevant time scales, defining a long-lived, metastable aggregated state. Importantly, self-limiting describes the progressive slowing of aggregation, whereas metastable describes the kinetic persistence of the resulting aggregated state. While particle–particle contacts have been shown to be effectively irreversible in the present system, this does not preclude the use of the term metastable, which here refers to the kinetic persistence of the aggregated state rather than to reversible particle association.

### 3.4 SERS Persistence, Proof-of-Concept Detection, and Reproducibility

To evaluate whether the bilastine-induced aggregates remain SERS-active over time, selected cit-AuNP/bilastine mixtures were analyzed by SERS. The temporal persistence and reproducibility of hotspots remain major challenges for colloidal SERS substrates, particularly when aggregation is induced by nonspecific electrolytes or rapidly acting aggregating agents. In such systems, continued aggregate growth and precipitation often lead to progressive loss of SERS activity.^15,31^ Because Raman microscopy probes a localized sampling volume, SERS intensity alone cannot provide a direct measurement of aggregate size, morphology, or colloidal stability. Nevertheless, persistence of the SERS response indicates that SERS-active aggregates and accessible hotspots remain present within the probed volume over the investigated time window.

For both bilastine concentrations tested, 0.4 and 1 μM, SERS signal persisted over several hours (Figure 4), confirming that SERS-active aggregates remain present during the monitored period. During the first hour, the overall SERS intensity increases for both samples, consistent with the progressive formation of plasmonically coupled aggregates and accessible interparticle hotspots. After 6 h, however, the two systems exhibit different behavior: the signal continues to increase for the 0.4 μM sample, whereas it decreases for the 1 μM sample. This difference is consistent with concentration-dependent aggregate evolution. At 1.0 μM bilastine, the lower magnitude of the ζ-potential and the continuous evolution of the lower-energy plasmon band suggest faster aggregation and structural reorganization on the hour time scale. Therefore, the decrease in SERS intensity after 6 h may arise from the formation of larger or partially sedimented aggregates that contribute less efficiently to the probed SERS volume. In contrast, the 0.4 μM system appears to remain closer to the desired slowly evolving regime, in which SERS-active interparticle junctions remain accessible for longer periods.

**Figure 4:**
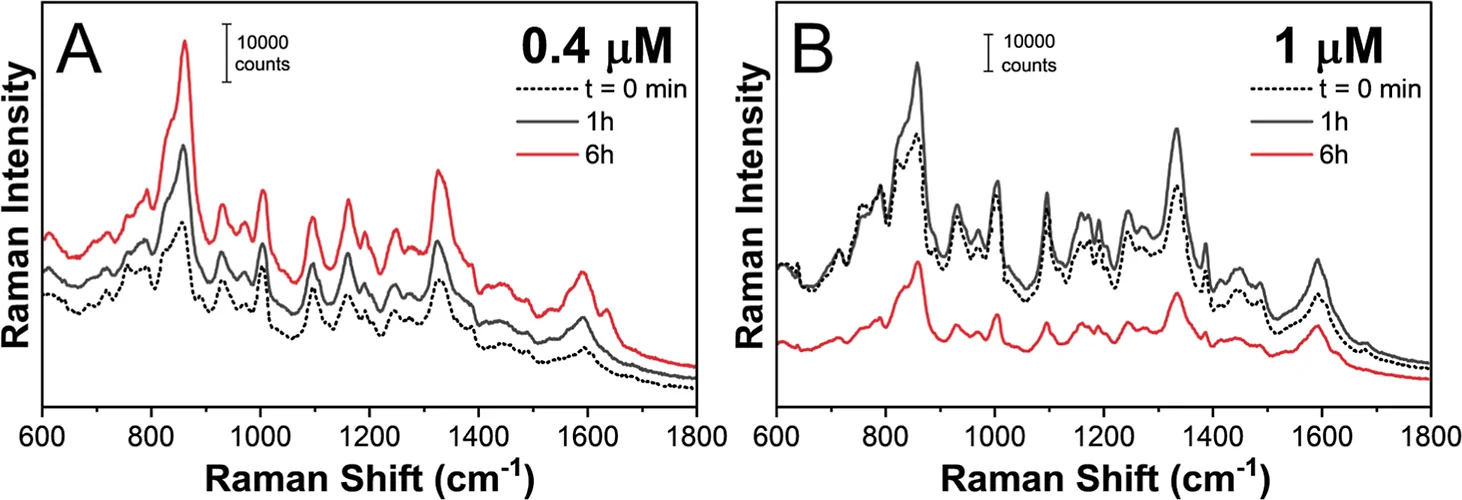
SERS spectra of cit-AuNP/bilastine mixtures containing (A) 0.4 μM and (B) 1 μM bilastine, recorded immediately after preparation, after 1 h and after 6 h.

The relationship between extinction and SERS intensity should also be interpreted cautiously. Although time-dependent extinction spectra show changes near the 785 nm excitation wavelength (Figure S10), ensemble optical extinction does not necessarily scale linearly with local SERS enhancement. Extinction includes absorption and scattering contributions from all suspended aggregates, including large or compact structures that may not generate efficient or accessible hotspots. SERS intensity, in contrast, depends on local electromagnetic enhancement, analyte location within hotspots, aggregate morphology, sedimentation, and the number of active aggregates within the laser sampling volume. Thus, the stronger extinction observed for more aggregated systems does not necessarily lead to proportionally stronger SERS signals. Similarly, shifts of plasmon band away from the excitation wavelength may influence the resonance condition but cannot alone explain the observed temporal changes in SERS intensity.^6,64^

Taken together, these results indicate that the most persistent SERS-active regime is obtained at submicromolar bilastine concentrations. Above this range, aggregation proceeds more rapidly, leading to structures that remain optically coupled but contribute less efficiently to the SERS response within the sampled volume. Therefore, the SERS data support the proposed dynamic self-limiting aggregation mechanism, while also showing that excessive bilastine concentrations accelerate aggregate evolution beyond the optimal hotspot regime.

As a proof-of-concept demonstration of whether these SERS-active aggregates persist with the addition of other analytes, bisphenol A (BPA) was introduced in the system as a model analyte (Section S.9, Supporting Information). When 0.15 μM bilastine was used as the aggregation modulator, the characteristic SERS band of BPA remained detectable for more than 30 days, with only a modest decrease in overall intensity. The suspension also remained visually stable during this period, retaining its characteristic color. Importantly, the spectral features associated with bilastine did not prevent identification of the main BPA bands, indicating that bilastine-induced aggregation can generate persistent SERS-active conditions in the presence of an additional analyte.

SERS reproducibility of the bilastine-induced aggregation was evaluated using 15 independently prepared cit-AuNP/bilastine samples at 0.15 μM bilastine. Monitoring the ν_s_(CO_2_^–^) mode yielded a relative standard deviation of 22.0% (Figure S12). This level of variability is typical for wet colloidal SERS substrates based on controlled aggregation and indicates reasonable batch-to-batch reproducibility under the present conditions. Further optimization of nanoparticle synthesis, bilastine concentration, incubation time, and sampling geometry may improve this performance.^15^

Finally, the bilastine concentration window identified here should be regarded as operational rather than universal. The aggregation kinetics are expected to depend on nanoparticle concentration, particle size, available surface area, collision frequency, ionic composition, and the bilastine-to-nanoparticle ratio.^7^ Therefore, the present results establish a useful framework for bilastine-mediated control of plasmonic aggregation, while future studies should examine how nanoparticle size and concentration affect the metastable SERS-active regime. Further investigation is required to assess the dependence of this behavior on nanoparticle size and concentration. Nevertheless, the present results provide a useful foundation for future studies of this interaction.

## 4 Conclusion

In summary, bilastine induces concentration-dependent aggregation of citrate-capped gold nanoparticles in aqueous media, with selected submicromolar concentrations producing slowly evolving metastable aggregates that persist over several hours to days. UV–Vis spectroscopy and DLS support the formation of plasmonically coupled aggregates in suspension, while conventional TEM provides qualitative evidence of concentration-dependent changes in dried state morphology. The combined ζ-potential, pH-dependent, and SERS results indicate that bilastine substantially modifies the citrate-stabilized interface, most likely through adsorption or co-adsorption, partial charge regulation, and perturbation of the interfacial ionic environment. Although complete citrate displacement and a unique bilastine adsorption geometry cannot be established from the present data, the results support a mechanism in which partially protonated bilastine reduces electrostatic repulsion and promotes nanoparticle association.

The aggregation process is best described as dynamically self-limiting rather than fully arrested. Time-dependent optical measurements show continued structural evolution, while semi-quantitative DLVO-type calculations rationalize the concentration-dependent reduction of the repulsive interaction barrier. At the same time, the limitations of the model indicate that additional non-DLVO interactions, including hydration, steric, hydrophobic, aromatic, and acid–base contributions, are likely involved. Once aggregates are formed, aged TEM images suggest that particle association is not readily reversible under the investigated conditions.

Importantly, the resulting aggregates retain SERS activity over extended periods, indicating the persistence of accessible SERS-active interparticle junctions within the sampled volume. The proof-of-concept BPA experiment and the reproducibility measurements further show that bilastine-mediated aggregation can provide temporally persistent SERS-active colloids with typical batch-to-batch reproducibility for wet colloidal substrates. Overall, these findings identify bilastine as a structurally multifunctional molecular modulator of plasmonic aggregation and suggest that pharmaceutical molecules with multiple ionizable and interfacially active groups may offer useful chemical handles for tuning colloidal SERS-active assemblies.

## Supplementary Material



## Notes

Cit-AuNPs size distribution, additional TEM images, DLS results, pH-dependent studies, theoretical DLVO description and parameters used in the DLVO estimations, control SERS spectra of cit-AuNPs, complete dataset for time-dependent UV–Vis experiments, proof-of-concept experiments of BPA detection and reproducibility experiments. This material is available free of charge (PDF)
