# Supplementary material for: Controlled Aggregation of Citrate-Stabilized Gold Nanoparticles into Long-Lived Metastable Clusters
Source: Langmuir. 2026 Sep 12;42(38):28105–13. doi: 10.1021/acs.langmuir.6c03197 (PMC13629677; doi:10.1021/acs.langmuir.6c03197)
Supplement: Supplementary file 1 [file la6c03197_si_001.pdf]

Supplementary Information

# Controlled Aggregation of Citrate-Stabilized Gold Nanoparticles into Long-Lived Metastable Clusters

Maelly A. Oliveira and Rômulo A. Ando*

*e-mail: raando@iq.usp.br

Laboratory of Molecular Spectroscopy, Department of Fundamental Chemistry,

Institute of Chemistry, University of São Paulo, Avenida Professor Lineu Prestes, 748,

São Paulo, SP, CEP 05508-000, Brazil

## Table of Contents

**S1. Cit-AuNPs size distribution** ........ 2

**S2. Additional TEM images** ........ 2

**S3. DLS results and interaction potential estimation** ........ 5

**S4. pH-dependent studies** ........ 6

**S5. DLVO Theory** ........ 7

**S6. Control SERS spectra** ........ 12

**S7. Aged samples** ........ 13

**S8. Time-dependent extinction: complete dataset** ........ 14

**S9. Bisphenol A detection** ........ 14

**S10. SERS Reproducibility** ........ 16

**References** ........ 16

## S1. Cit-AuNPs size distribution

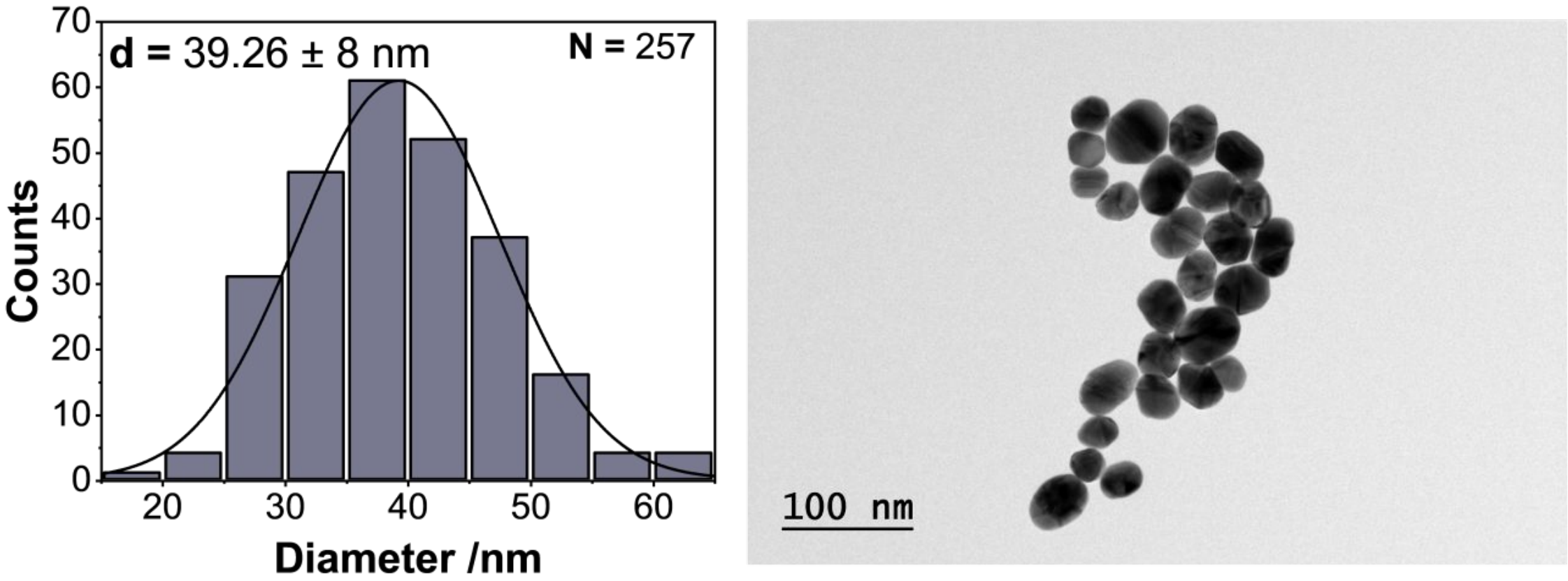


Figure S1. Cit-AuNP size distribution obtained from TEM images with N = 257.

## S2. Additional TEM images

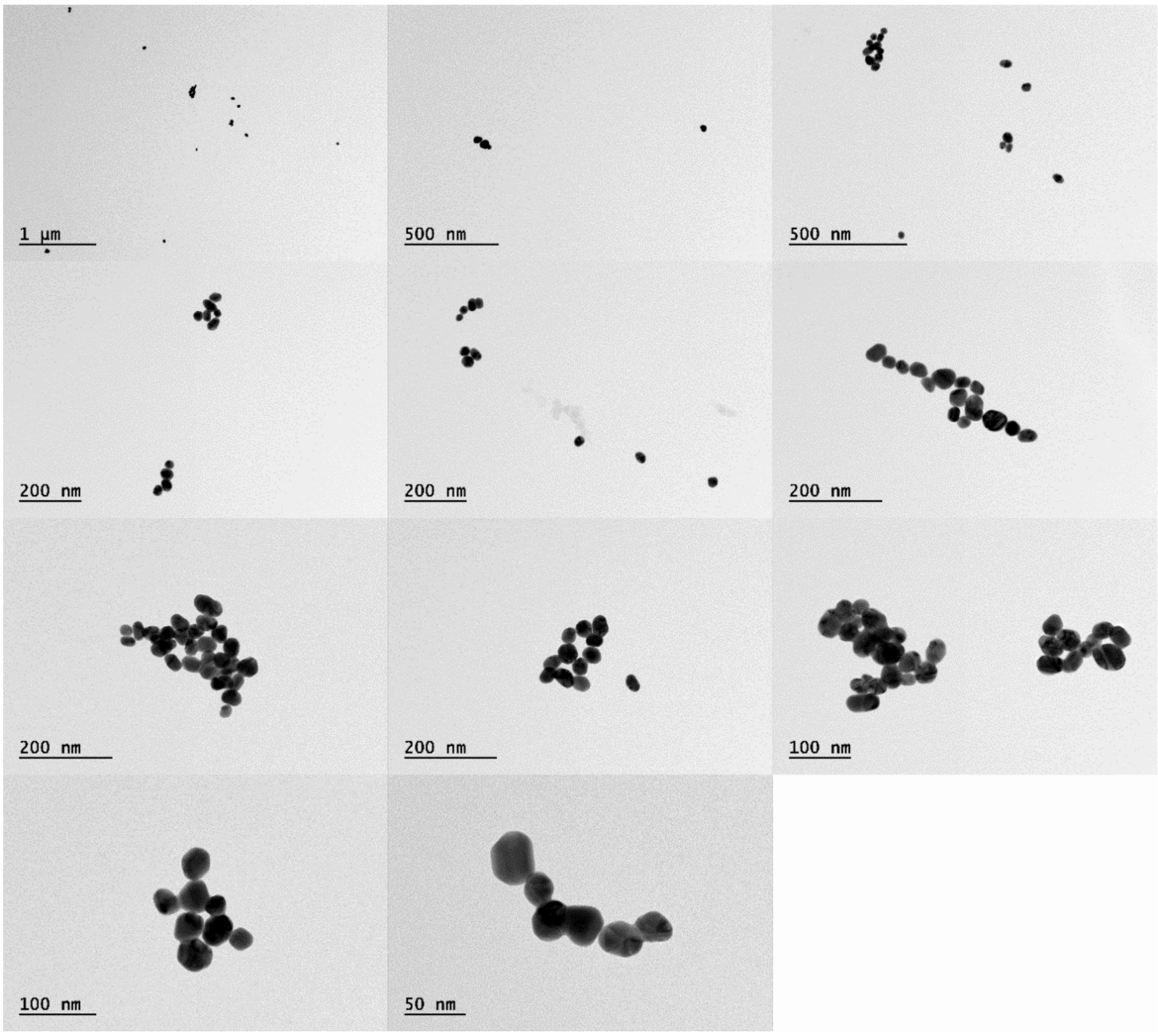

**Figure S2.** Representative TEM images of cit-AuNP/bilastine mixtures at **[BIL] = 0.4 μM**.

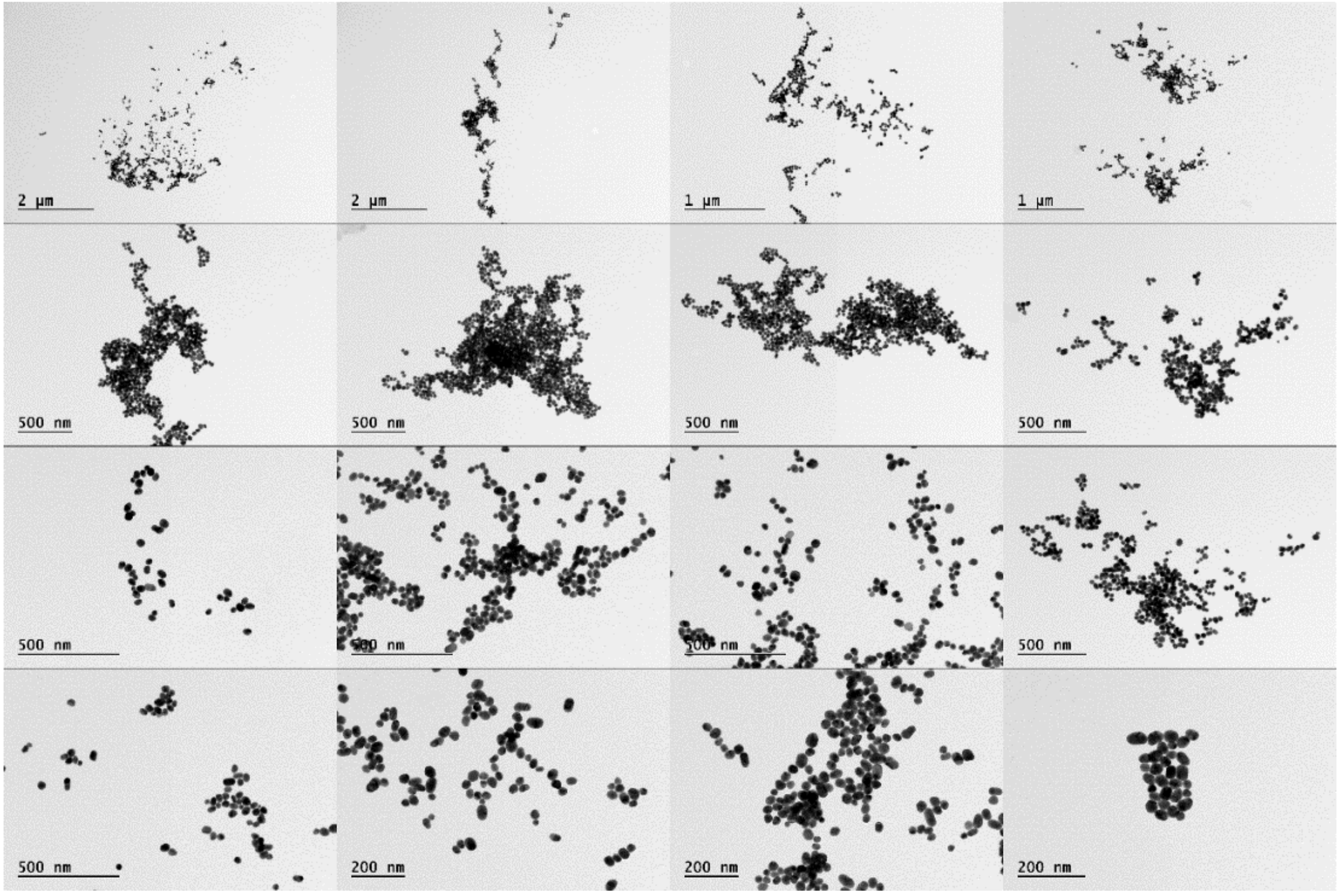


**Figure S3.** Representative TEM images of cit-AuNPs/bilastine mixtures at **[BIL] = 0.8 µM**.

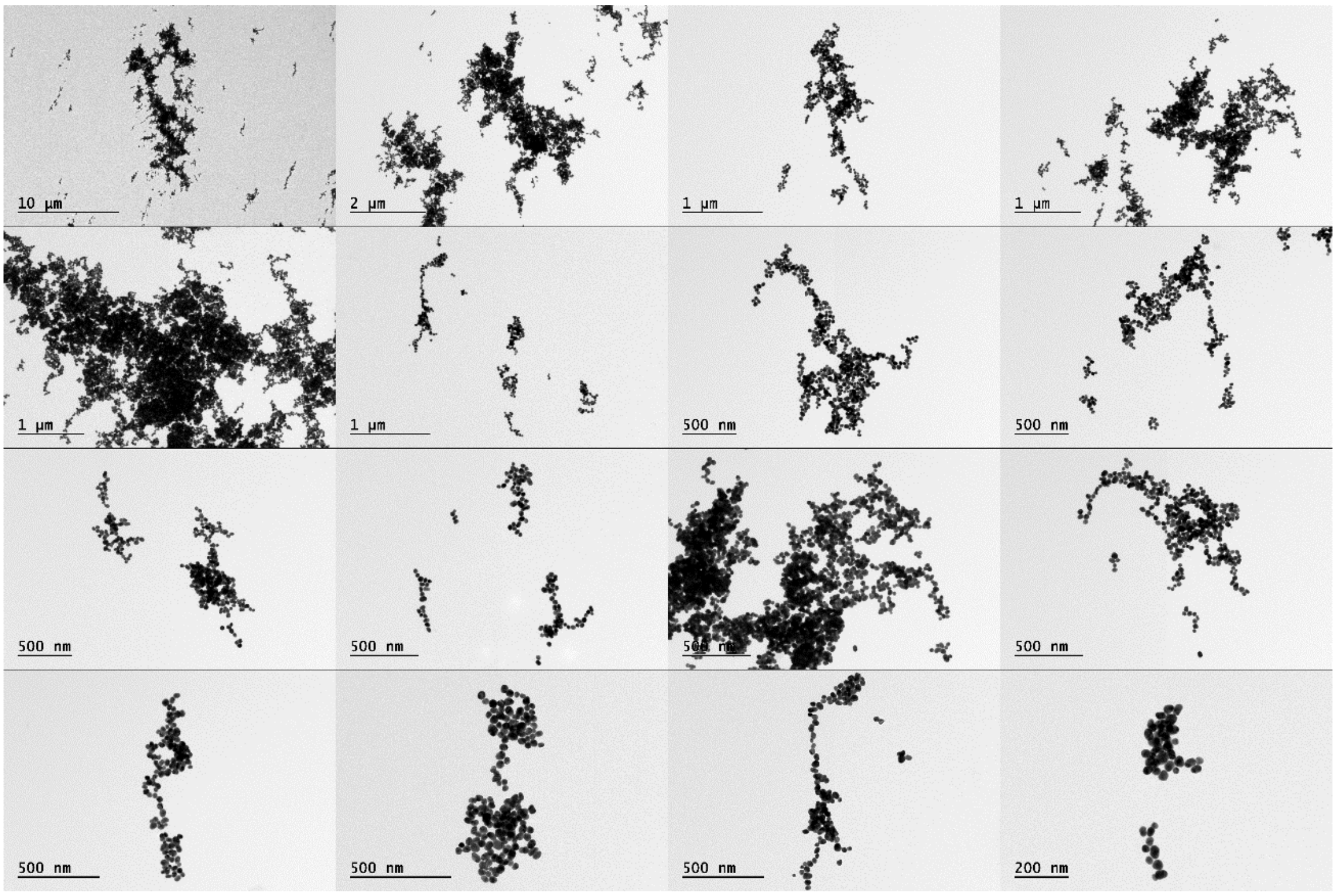


**Figure S4.** Representative TEM images of cit-AuNPs/bilastine mixtures at **[BIL] = 2 µM**.

## S3. DLS results and interaction potential estimation

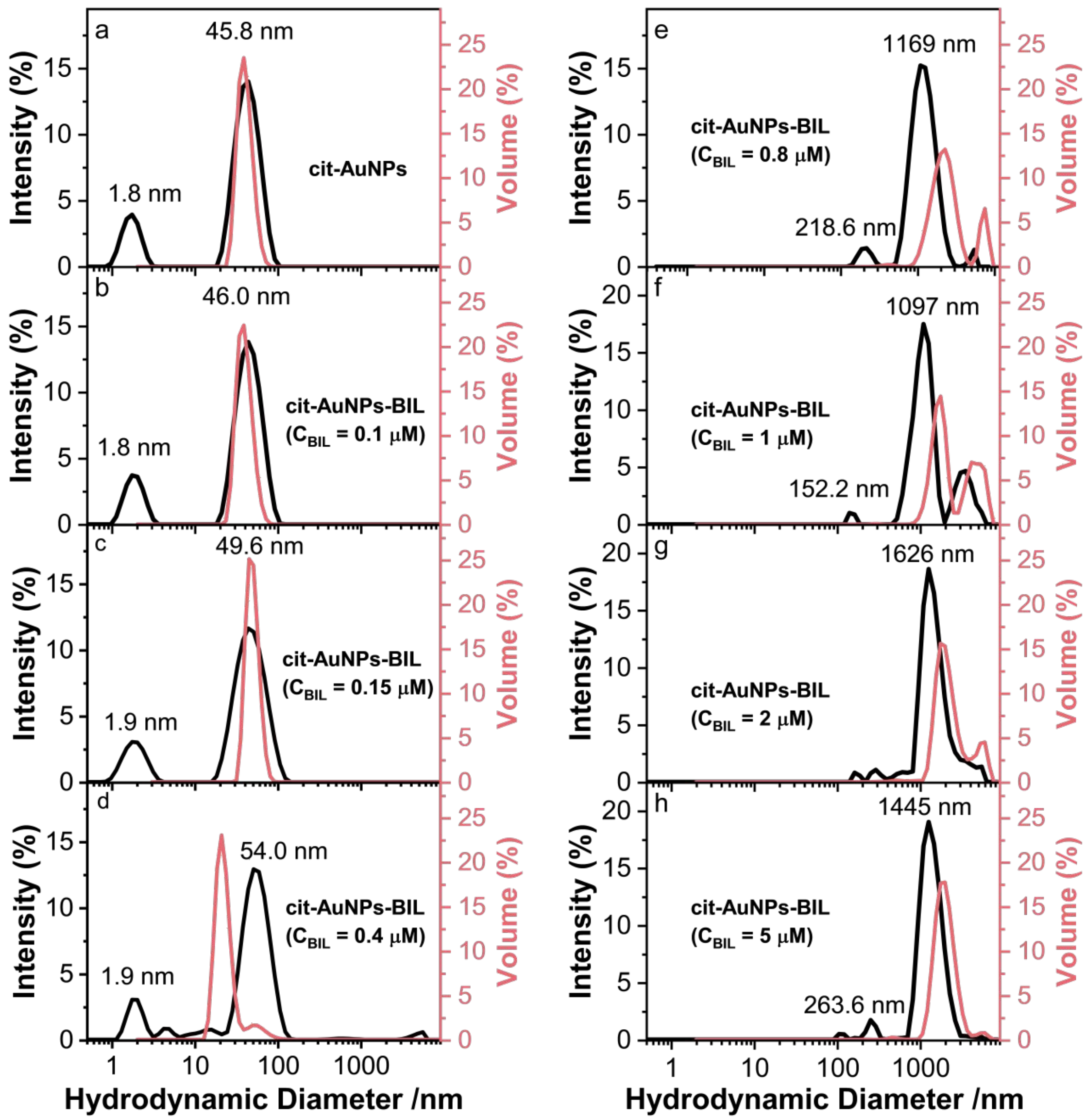


**Figure S5**. Dynamic Light Scattering measurements (intensity-weighted and volume weighted) of selected cit-AuNP-bilastine mixtures: (a) pure cit-AuNP, (b) 0.1 μM bilastine, (c) 0.15 μM, (d) 0.4 μM, (e) 0.8 μM, (f) 1.0 μM, (g) 2 μM, (h) 5 μM.

### S4. pH-dependent studies

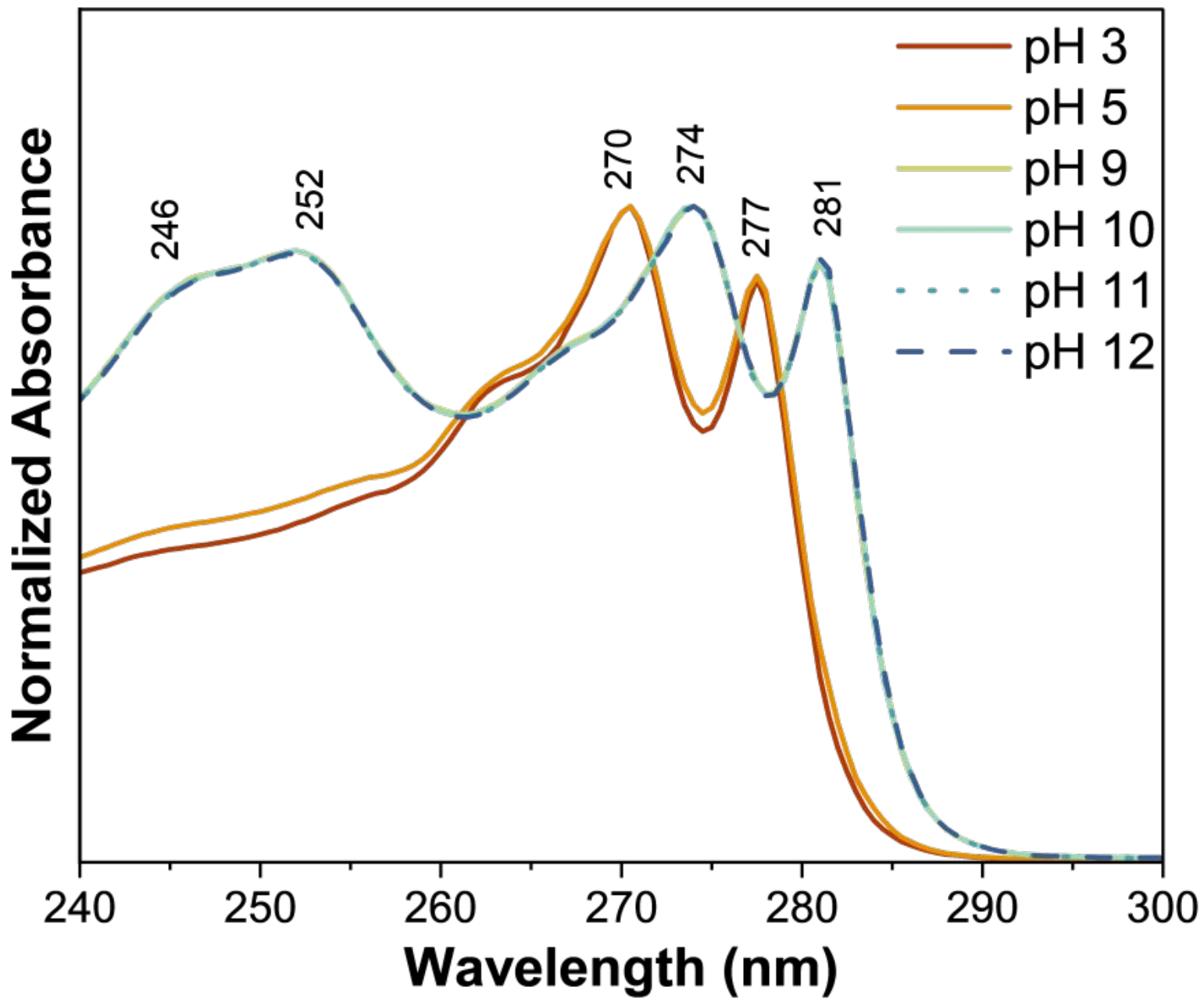


**Figure S6.** Extinction spectra of an aqueous 0.5 mM BIL solution obtained in different pHs. The pH was adjusted using 0.1 M HCl or 0.1 M NaOH as required.

The electronic spectrum of an aqueous 0.5 mM bilastine solution is shown in Fig. S6. The most prominent bands at 274 and 281 nm are assigned to $\pi \rightarrow \pi^*$ transitions of aromatic chromophores, whereas the bands at 246 and 252 nm are associated with the benzimidazole moiety, in agreement with reported spectra of benzimidazole derivatives.[1]

At pH ≤ 5, the lower energy bands undergo a small blueshift, while the higher energy bands become less resolved, indicating that protonation affects the benzimidazole chromophore. Literature $pK_a$ values are available for carboxylic acid (pKa = 4,15 ± 0.06),[2] and for the piperidine moiety (pKa = 9.43),[3,4] whereas the protonation behavior of the benzimidazole unit in aqueous bilastine is less clearly established. The present pH-dependent UV-Vis data suggest that the benzimidazole moiety contributes to the overall

protonation equilibrium, with an apparent spectral transition occurring between pH 5 and 7.

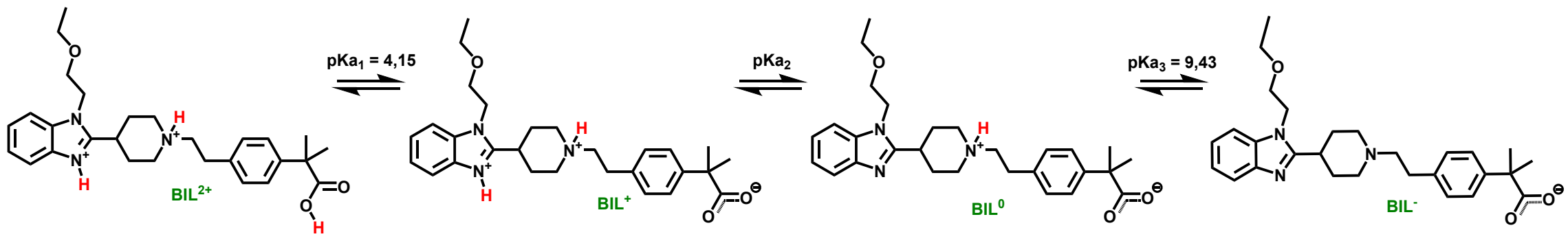


**Scheme S1.** Suggested updated equilibria for Bilastine, based on the results obtained via electronic spectroscopy and pKa values from literature.

The observed blueshift in low pH can be rationalized by protonation of the benzimidazole nitrogen, which modifies electron delocalization within the aromatic heterocycle. Protonation reduces the contribution of the nitrogen lone pair to the conjugated system and shifts the corresponding π→π* transitions to higher energy.[1]

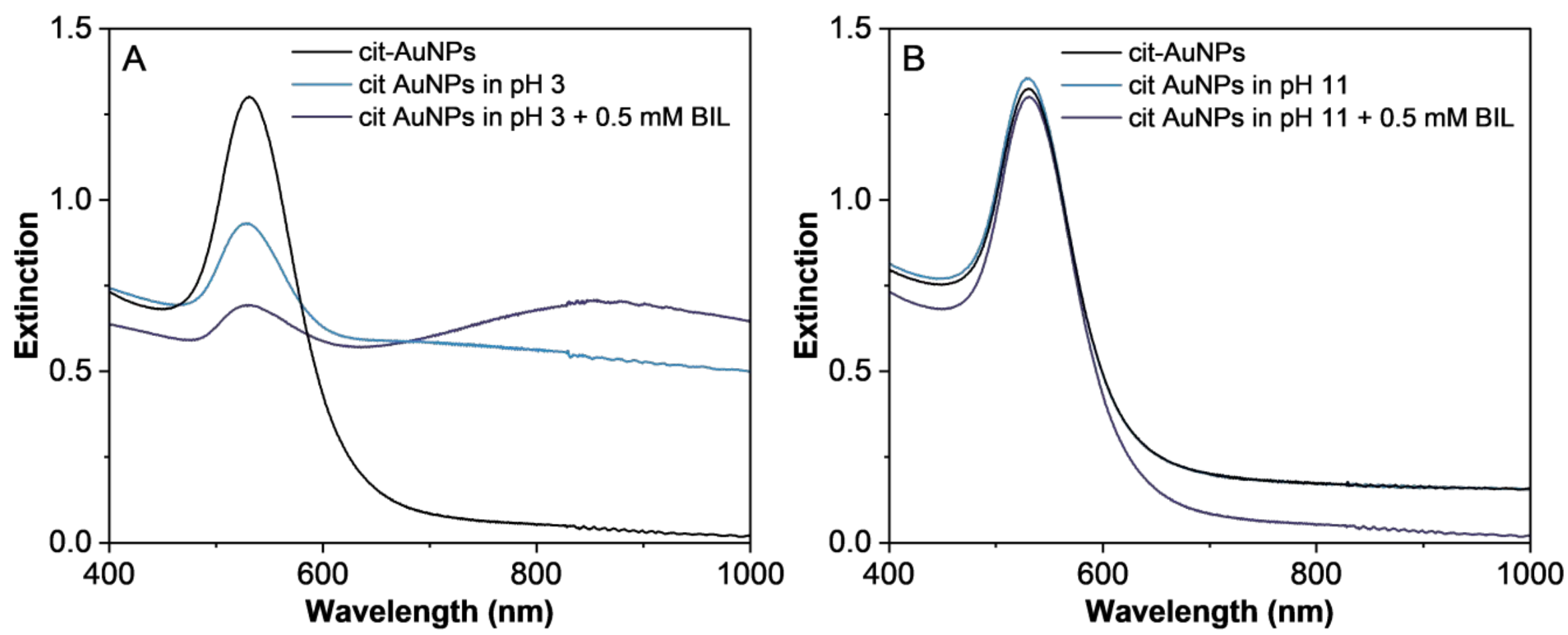


**Figure S7**. Extinction spectra of cit-AuNP-bilastine mixtures **(A)** in pH 3 and **(B)** in pH 11.

## S5. DLVO Theory

Within the extended DLVO framework employed in this work, the total interaction potential ($U_T$) between two spherical particles in aqueous medium can be

primarily approximated as the sum of the van der Waals attractive interaction ($U_{vdW}$), the electrostatic repulsion ($U_e$) and Born-repulsive interactions ($U_{Born}$).[6–9] The attractive van der Waals contribution favors particle association and represents the negative contribution to the total interaction potential. Attractive interactions between two particles with identical radius immersed in a medium can be calculated as:

$$\frac{U_{vdW}}{k_B T} = -\left(\frac{A}{6k_B T}\right)\cdot\left(\frac{2r^2}{h^2 + 4rh} + \frac{2r^2}{(h + 2r)^2} + \ln\left(\frac{h^2 + 4rh}{(h + 2r)^2}\right)\right) \quad (1)$$

where $r$ is the particle or cluster radius, $h$ is the edge-to-edge interparticle distance, $T$ is the absolute temperature, $k_B$ is the Boltzmann constant and A is the Hamaker constant for a pair of gold nanoparticles interacting through an aqueous medium. [9,10]This constant is size-dependent, and its reported value varies significantly across the literature. According to models developed by Wijenayaka *et al.*, a Hamaker constant of $3 \times 10^{-19}$ J reasonably represents the systems investigated here and, therefore, this value was maintained for all subsequent DLVO calculations.[10]

Conversely, electrostatic interactions are modeled according to the electric double layer (EDL) theory, accounting for ions adsorbed and accommodated within a diffuse layer. This interface is characterized by particle surface potential ($\psi_0$) and the Debye length ($\kappa^{-1}$), a parameter governed heavily by the ionic strength and bulk electrolyte concentration. Total electrostatic interaction for particles with identical radii $r$considering the edge-to-edge separation $h$ is given by:

If $\kappa r < 5$ (double layer thickness is small compared to the particle radius)

$$\frac{U_{ele}}{k_B T} = \frac{4\pi\epsilon\epsilon_0 r^2 Y^2 k_B T}{e^2}\left(\frac{\exp(-\kappa h)}{h + 2r}\right)(2)$$

If $\kappa r > 5$ (double layer thickness is large compared to the particle radius)

$$\frac{U_{ele}}{k_B T} = \frac{2\pi\epsilon_0\epsilon\varphi_0^2 r}{k_B T}\cdot \ln(1 + \exp(-\kappa h))(3)$$

$$Y = \frac{8\tanh\left(\frac{e\varphi_0}{4k_B T}\right)}{1 + \left(1 - \frac{2\kappa r + 1}{(\kappa r + 1)^2} + \left(\frac{e\varphi_0}{4k_B T}\right)\right)^{1/2}}(4)$$

$$\kappa = \sqrt{\frac{2e^2 N_A I}{\epsilon\epsilon_0 k_B T}}(5)$$

Where $\varphi_0$ is the surface potential, $\kappa$ is the inverse Debye length, $I$ is the ionic strength of the medium, $\epsilon_0$ is the vacuum permittivity, $\epsilon$ is the relative permittivity of the solution (78.5 for water), $e$ is the electron charge, and $N_A$ is the Avogadro constant. The ionic strength was kept constant in the calculations because the submicromolar bilastine concentrations used here are negligible relative to nominal millimolar ionic background of the as-synthesized colloid.

Usually, the surface potential $\varphi_0$ is approximated by the experimentally measurable $\zeta$-potential. However, this approximation is reasonable if the ionic strength of the solution is high enough that the Debye length ($\kappa^{-1}$) is small compared to the relevant separation distances evaluated in the potential curve. Considering the low ionic strength of the system in investigation (~3.4 mM), such approximation could lead to a significant underestimation of the magnitude of the electrostatic interaction. According to Wijenayaka *et al.*, it is reasonable to estimate $\varphi_0$ as Eq. (6), which assumes that the

dielectric constant around the particles is independent of the radial distance from the surface, and that the energy required to transport charges in solution depends solely on coulombic interactions.

$$\varphi_0 = \zeta\left(1 + \frac{1}{\kappa r}\right) \cdot \exp(1) \quad (6)$$

In addition to the electrostatic repulsion arising from the resulting positive charges, bilastine molecules adsorbed onto the nanoparticle surface can introduce short-range repulsive forces to the estimated total potential, representing non-DLVO forces such as steric and hydration forces. Because specific parameters for a bilastine adsorbed layer, such as layer thickness and surface coverage, are not available, the Born-type short-range repulsive term was included only as generic correction to avoid an unphysical divergence of the primary minimum at very short separations. The formalism developed by Feke *et al.* is appropriate for adsorption phenomena, and can be expressed as follows:

$$\frac{U_{Born}}{k_B T} = \frac{4A}{k_B T}\left(\frac{\sigma_c}{r}\right)^6 \left(\frac{4!}{10!}\right)\left(\frac{1}{R}\right)\left(\frac{R^2 - 14R + 54}{(R-2)^7} + \frac{-2R^2 + 60}{R^7} + \frac{R^2 + 14R + 54}{(R+2)^7}\right) \quad (7)$$

where $R = \frac{h+2r}{r}$ and $\sigma_c$ is the collision diameter, with typically equal to 0.5 nm.[11,12]

Finally, the total interaction potential, in $k_B T$ units, can be obtained considering Eq. (8):

$$U_T = \frac{U_{vdW}}{k_B T} + \frac{U_{ele}}{k_B T} + \frac{U_{Born}}{k_B T} \quad (8)$$

The resulting interaction potential can be represented as a function of the edge-to-edge interparticle distance (*h*). At intermediate separations, a significant energy barrier arises due to electrostatic repulsion between the negatively charged citrate layers

surrounding each particle of a citrate-capped gold colloid. At shorter distances, however, this barrier is overcome, and the potential energy decreases sharply as vdW attractions dominate, leading to irreversible aggregation corresponding to the thermodynamically favored state. Accordingly, the magnitude of this energy barrier is a key determinant of colloidal stability: the higher the barrier, the more kinetically stable the colloid. The propensity and kinetics of nanoparticle aggregation are therefore directly related to the reduction of this energy barrier, which facilitates particle approach.

**Table S1.** Parameters used for the semi-quantitative DLVO-type interaction potential estimates. Ionic species concentrations were estimated from the initial synthetic composition and should be regarded as nominal values.

| | Concentration of bilastine (μM) | | | | |
|---|---|---|---|---|---|
| | 0 | 0.10 | 0.15 | 0.4 | 0.8 |
| **Zeta potential (mV)** | -29.3 | -32.3 | -34.4 | -21.0 | -16.5 |
| **Hydrodynamic Diameter (nm)** | 45.8 | 46.0 | 49.6 | 54.0 | 218.6 |
| **Ionic Strength (mM)** | 3.4 | 3.4 | 3.4 | 3.4 | 3.4 |

*Concentration of species in solution (mM)*[a]

| [b]$[HCit]^{2-}$ | $[Cl]^-$ | $[Na]^+$ |
|---|---|---|
| 0.228 | 2.43 | 3.42 |

[a] Concentrations estimated from initial reactants of the Turkevich synthesis.

[b] Considering the pH of the cit-AuNPs (pH = 5), the major species in solution is the singly protonated citrate anion.

**S6. Control SERS spectra**

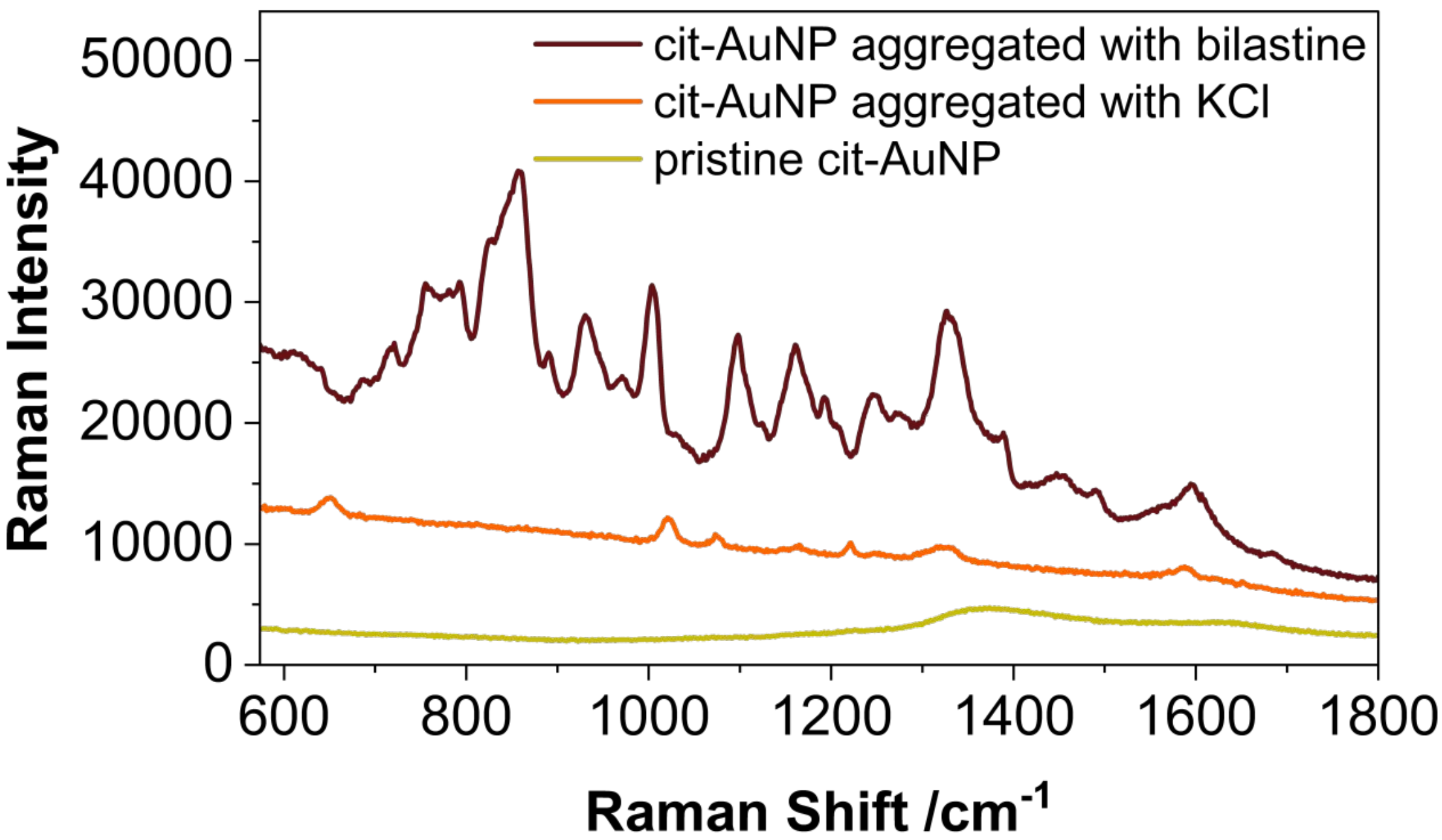


**Figure S8.** Control comparison of SERS spectra obtained from pristine cit-AuNPs (beige), KCl-aggregated cit-AuNPs (orange), and bilastine-aggregated cit-AuNPs (brown). Solutions with final concentrations of 0.2 mM for KCl and 0.4 μM for bilastine were used.

## S7. Aged samples

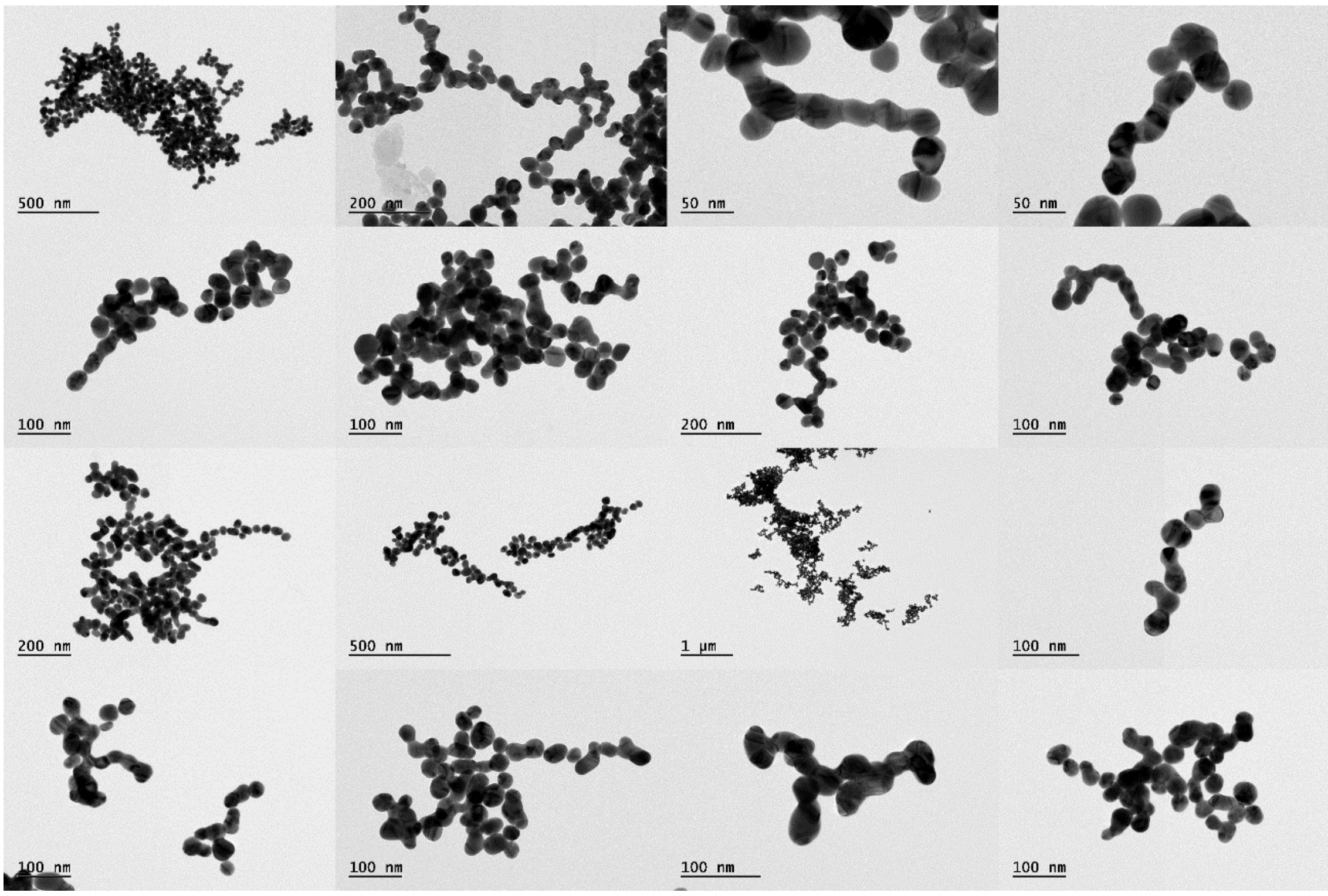


**Figure S9.** TEM images of an aged sample of cit-AuNP when **[BIL] = 0.8 μM**.

A freshly prepared cit-AuNPs/bilastine mixture containing 0.8 μM bilastine was stored for 1 month and then prepared for TEM using the procedure described in the Experimental Section of the main text. The images show large, irregular aggregates together with elongated assemblies. The apparent particle-particle attachment is consistent with the progressive structural evolution of aggregates during prolonged aging. During prolonged storage, nanoparticles that remain in close contact within aggregates can undergo interfacial rearrangement, formation of stronger particle-particle contacts, and partial coalescence, driven by reduction of interfacial surface energy.

Because conventional TEM requires drying, these images should be interpreted as qualitative evidence of structural evolution after prolonged storage rather than as a direct representation of the solution-phase aggregate distribution.

## S8. Time-dependent extinction: complete dataset

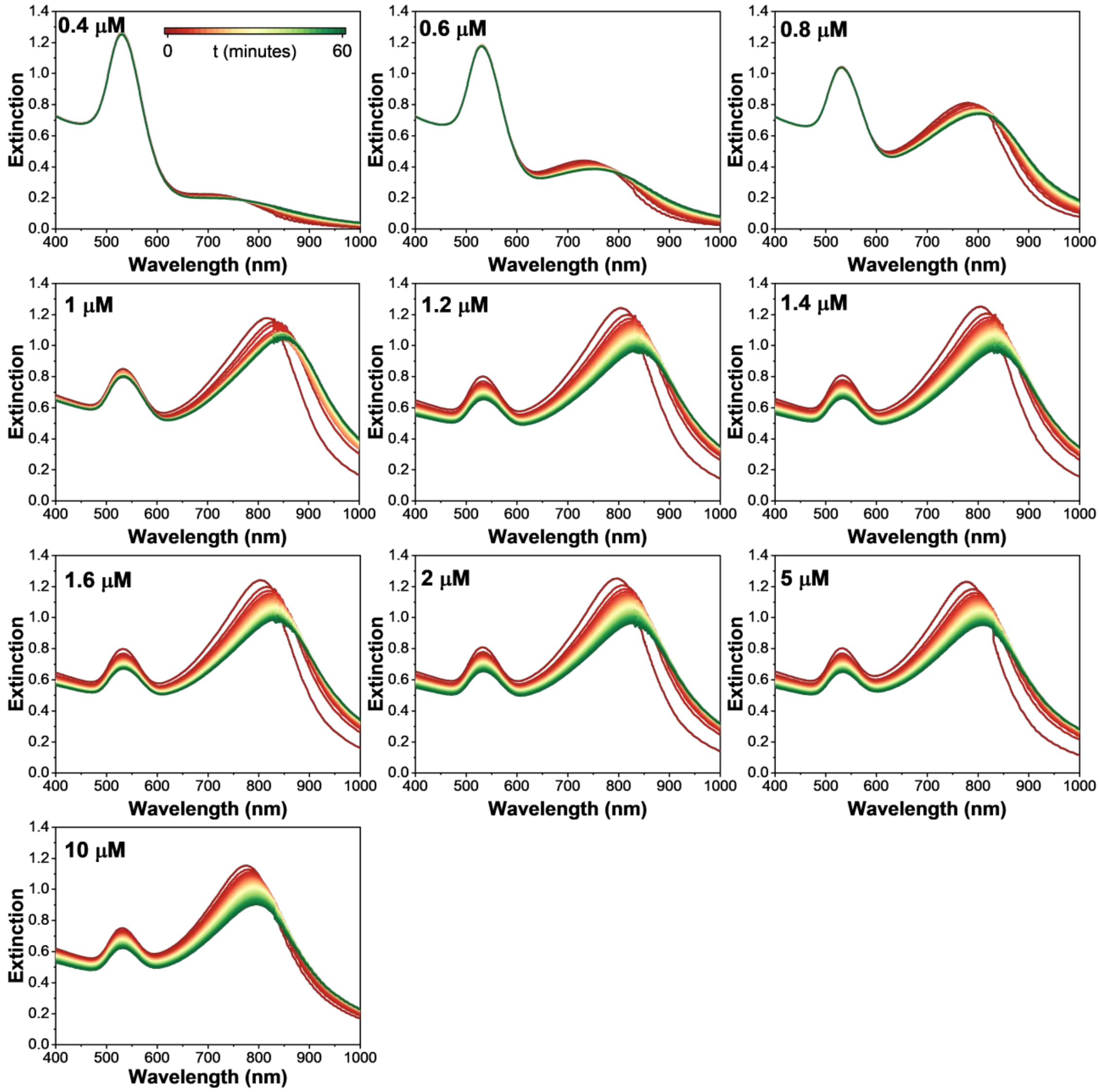


Figure S10. Complete dataset for time-dependent (0 to 60 min) extinction spectra of cit-AuNP after the addition of 0.4 to 10 μM BIL.

## S9. Bisphenol A detection

To evaluate whether additional compounds interfere with the aggregation process and the practical utility of the proposed SERS platform, samples were prepared by adding 20 μL of methanolic bisphenol A (BPA, 0.2 mM) and 20 μL of aqueous bilastine (0.15 μM)

to 960 μL of gold colloid. For comparison, a control sample was prepared by replacing bilastine with 20 mM KCl.

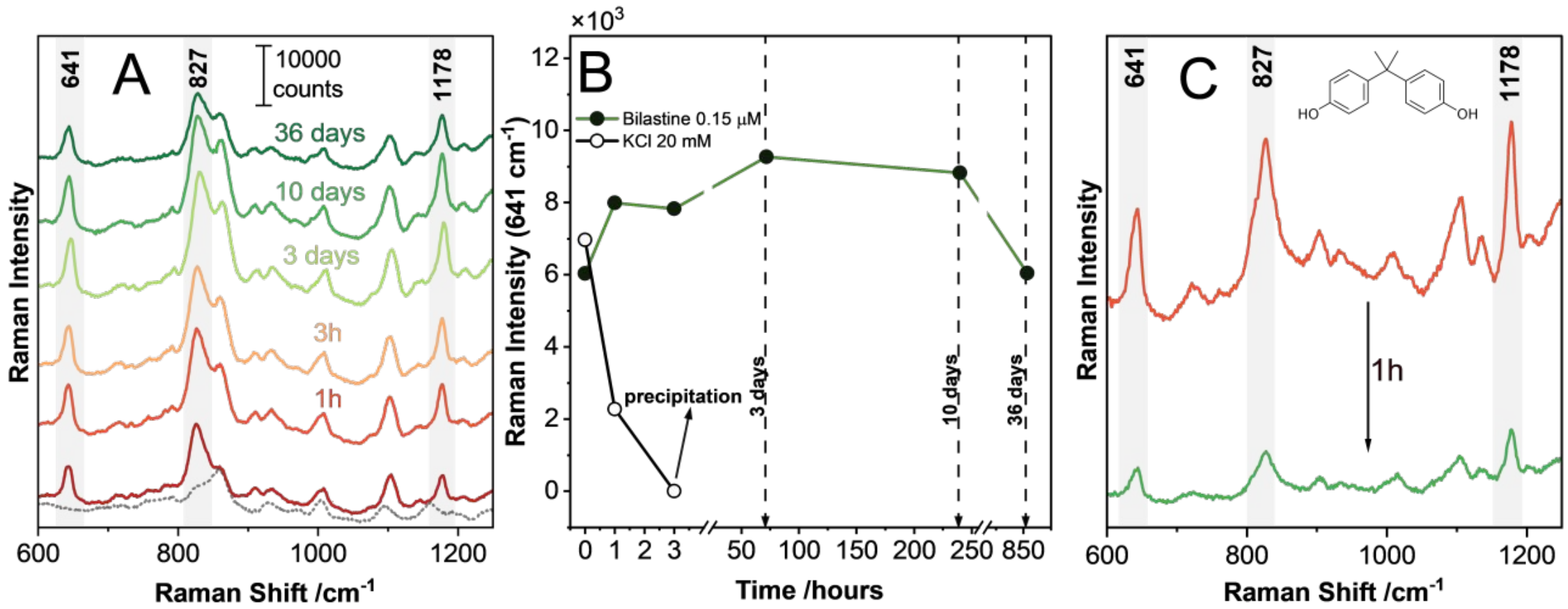


**Figure S11.** (A) SERS spectra of a 4 μM BPA sample monitored for 36 days. Data was plotted offset for clarity. Dotted grey line: "Blank" spectrum of bilastine on the same scale. (B) Time-dependent absolute intensity of the 641 $cm^{-1}$ BPA mode with bilastine or KCl as aggregating agent. (C) SERS spectrum of a 0.2 mM BPA sample aggregated with KCl and the same sample after 1 hour. *Inset:* Molecular structure for BPA.

In the presence of bilastine, the SERS intensity of BPA-related bands (641, 827, and 1178 $cm^{-1}$)[13,14] remained stable for over 30 days. In contrast, although the KCl-aggregated control sample initially exhibited higher intensity, it demonstrated poor stability, with the signal decaying significantly after only 1 hour. The absence of significant shifts or spectral changes in the characteristic BPA bands suggests that bilastine does not substantially interfere with BPA identification under these conditions.

## S10. SERS Reproducibility

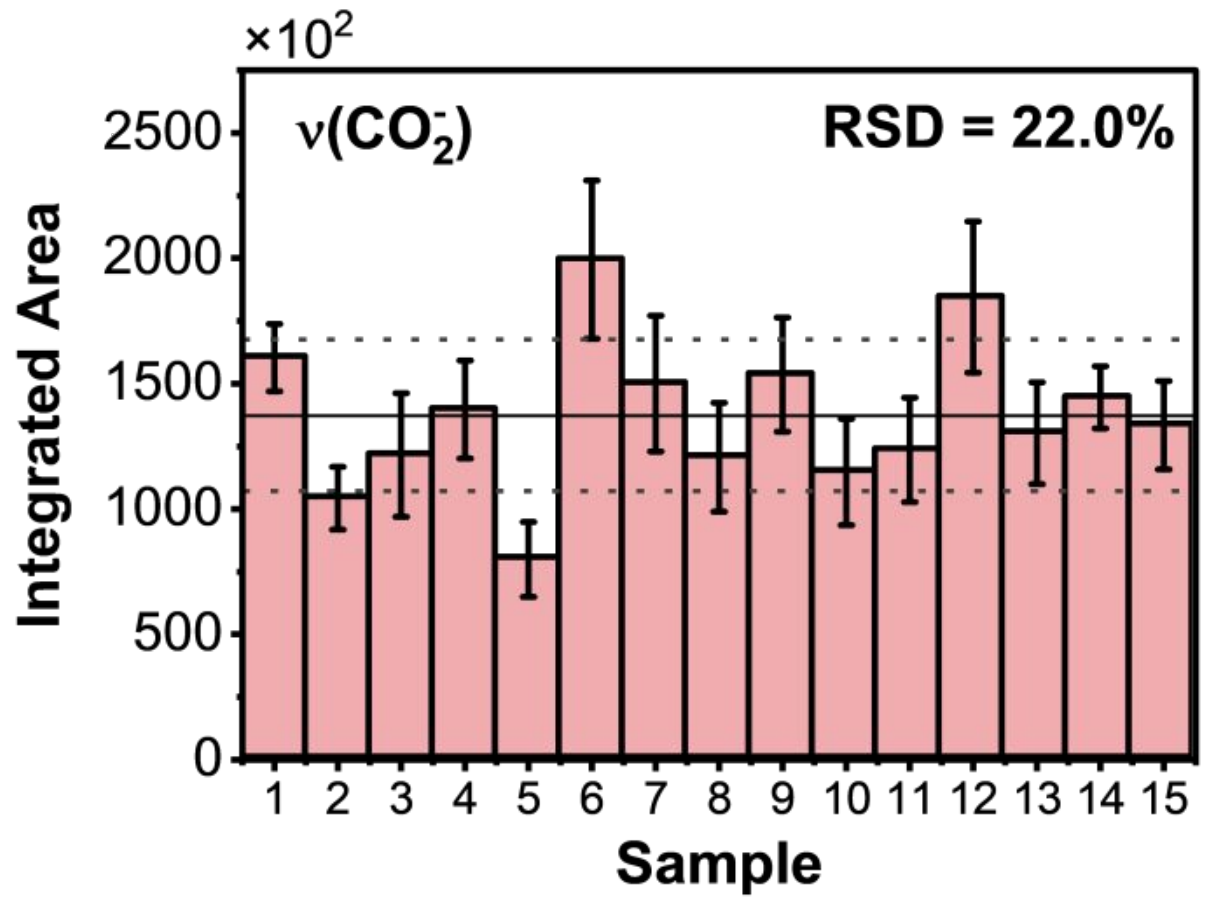


**Figure S12.** Reproducibility test monitoring the symmetric stretching vibrational mode of the carboxylate group.

### References


(1) Krishnamurthy, M.; Phaniraj, P.; Dogra, S. K. Absorptiometric and Fluorimetric Study of Solvent Dependence and Prototropism of Benzimidazole Homologues. *J. Chem. Soc., Perkin Trans. 2* **1986**, No. 12, 1917. https://doi.org/10.1039/p29860001917.

(2) PrBLEXTEN®.

(3) Kar, A.; Giri, L.; Kenguva, G.; Kumar Bommaka, M.; Bandi, S.; Dandela, R. Multi-Component Forms of the 2nd Generation H1 Receptor Antagonist Drug, Bilastine and Its Enhanced Physicochemical Characteristics. *Bioorganic Chemistry* **2024**, *151*, 107672. https://doi.org/10.1016/j.bioorg.2024.107672.

(4) Kawauchi, H.; Yanai, K.; Wang, D.-Y.; Itahashi, K.; Okubo, K. Antihistamines for Allergic Rhinitis Treatment from the Viewpoint of Nonsedative Properties. *IJMS* **2019**, *20* (1), 213. https://doi.org/10.3390/ijms20010213.

(5) University of Belgrade, Faculty of Pharmacy, Department of Pharmaceutical Chemistry, Serbia; Popović-Nikolić, M. R.; Popović, G.; University of Belgrade, Faculty of Pharmacy, Department of General and Inorganic Chemistry, Serbia; Oljačić, S.; University of Belgrade, Faculty of Pharmacy, Department of Pharmaceutical Chemistry, Serbia; Nikolić, K.; University of Belgrade, Faculty of

Pharmacy, Department of Pharmaceutical Chemistry, Serbia. Theoretical and Experimental Study of Bilastine Ionization. In *Book of Proceedings*; Institute for Information Technologies, University of Kragujevac, 2023; pp 427–430. https://doi.org/10.46793/ICCBI23.427PN.

(6) Zhang, W. Nanoparticle Aggregation: Principles and Modeling. In *Nanomaterial*; Capco, D. G., Chen, Y., Eds.; Advances in Experimental Medicine and Biology; Springer Netherlands: Dordrecht, 2014; Vol. 811, pp 19–43. https://doi.org/10.1007/978-94-017-8739-0_2.

(7) Kontogeorgis, G. M.; Kiil, S. *Introduction to Applied Colloid and Surface Chemistry*, 1st ed.; Wiley, 2016. https://doi.org/10.1002/9781118881194.

(8) Verwey, E. J. W. Theory of the Stability of Lyophobic Colloids. *J. Phys. Chem.* **1947**, *51* (3), 631–636. https://doi.org/10.1021/j150453a001.

(9) *Principles of Colloid and Surface Chemistry, Revised and Expanded*, 0 ed.; Hiemenz, P. C., Rajagopalan, R., Eds.; CRC Press, 2016. https://doi.org/10.1201/9781315274287.

(10) Wijenayaka, L. A.; Ivanov, M. R.; Cheatum, C. M.; Haes, A. J. Improved Parametrization for Extended Derjaguin, Landau, Verwey, and Overbeek Predictions of Functionalized Gold Nanosphere Stability. *J. Phys. Chem. C* **2015**, *119* (18), 10064–10075. https://doi.org/10.1021/acs.jpcc.5b00483.

(11) Liu, Y.; Han, X.; He, L.; Yin, Y. Thermoresponsive Assembly of Charged Gold Nanoparticles and Their Reversible Tuning of Plasmon Coupling. *Angew Chem Int Ed* **2012**, *51* (26), 6373–6377. https://doi.org/10.1002/anie.201201816.

(12) Feke, D. L.; Prabhu, N. D.; Mann, J. A.; Mann, J. A. A Formulation of the Short-Range Repulsion between Spherical Colloidal Particles. *J. Phys. Chem.* **1984**, *88* (23), 5735–5739. https://doi.org/10.1021/j150667a055.

(13) De Souza, M. L.; Valdivia, S.; Otero, J. C.; López-Tocón, I. Sensing Bisphenol A by Means of Surface-Enhanced Raman Spectroscopy and DFT Calculations to Elucidate the Enhancement Mechanism That Dominates the Spectrum. *Chemosensors* **2023**, *11* (2), 78. https://doi.org/10.3390/chemosensors11020078.

(14) Roschi, E.; Gellini, C.; Ricci, M.; Sanchez-Cortes, S.; Focardi, C.; Neri, B.; Otero, J. C.; López-Tocón, I.; Smulevich, G.; Becucci, M. Surface-Enhanced Raman Spectroscopy for Bisphenols Detection: Toward a Better Understanding of the Analyte–Nanosystem Interactions. *Nanomaterials* **2021**, *11* (4), 881. https://doi.org/10/gtsw7p.
